# Combined Animal and Plant Protein Enable High Fishmeal Replacement in *Penaeus vannamei* Under Biofloc Technology

**DOI:** 10.3390/cimb48090904

**Published:** 2026-09-04

**Authors:** S. Ferrando-Juan, J. Gómez-Aguilera, A. Tomás-Vidal, M. Rodilla, M. Jover-Cerdá, F. J. Moyano, D. S. Peñaranda, S. Martínez-Llorens

**Affiliations:** 1 Aquaculture and Biodiversity Research Group, Institute of Science and Animal Technology (ICTA), Universitat Politècnica de València, Camí de Vera, s/n, 46022 València, Spain; sferjua@upv.es (S.F.-J.); jgomagu@posgrado.upv.es (J.G.-A.); atomasv@dca.upv.es (A.T.-V.); mjover@dca.upv.es (M.J.-C.); dasncpea@upv.es (D.S.P.); 2 Research Institute for Integrated Management of Coastal Areas, Universitat Politècnica de València, Carretera de Nazaret-Oliva, s/n, Grao de Gandía, 46730 València, Spain; mrodilla@hma.upv.es; 3 Department of Biology and Geology, University of Almeria, Agrifood Campus of International Excellence CeiA3, CIAIMBITAL, Carretera Sacramento, s/n, La Cañada, 04120 Almeria, Spain; fjmoyano@ual.es

**Keywords:** fishmeal, animal protein, plant protein, nutrient utilization, shrimp microbiome, biofloc technology

## Abstract

The increasing demand and limited availability of fishmeal (FM) have intensified the need for sustainable alternative protein sources in shrimp aquafeeds. This study evaluated high FM replacement in juvenile *Penaeus vannamei* reared under biofloc technology (BFT), integrating in vitro protein digestibility, growth performance, nutrient utilization and microbiome analyses. Six isoproteic and isolipidic diets combined two FM levels (0% and 7.5%) with animal (A), plant (P), or mixed (AP) protein sources, plus a 15% FM Control diet were assayed. Plant-based diets showed higher in vitro protein digestibility and amino acid release than animal-based diets. However, these differences were not reflected in shrimp performance, as final body weight, SGR and survival were similar among dietary treatments. Apparent feed conversion ratio, protein efficiency, and economic performance were improved in shrimp fed the A7.5 and AP7.5 diets, whereas complete FM replacement resulted in poorer performance. Biofloc and shrimp intestinal microbial community composition was primarily driven by temporal succession, whereas dietary protein source modulated the abundance of specific bacterial families. Biofloc and intestinal communities shared limited taxonomic overlap at the family level, suggesting restricted microbial exchange together with strong habitat and host mediated selection. Overall, these findings indicate that a diet containing 7.5% fishmeal (FM), supplemented with either animal-derived proteins or a combination of animal- and plant-derived protein sources, represents an optimal strategy for juvenile *Penaeus vannamei* cultured under BFT conditions, maintaining performance while preserving intestinal microbial stability and health.

## 1. Introduction

Fishmeal (FM) has historically been the main protein source in aquafeeds due to its high digestibility, balanced amino acid (AA) profile, nutritional value, and palatability [1]. Nevertheless, limited availability and increasing demand have raised feed costs and intensified the search for sustainable alternatives [2]. Aquafeed formulation must therefore reduce dependence on marine resources while preserving nutritional and economic viability. This is particularly relevant for *Penaeus vannamei* (*P. vannamei*, Boone, 1931), whose dietary protein requirements generally range from 30% to 50% crude protein (CP), depending on growth stage and rearing conditions [3,4]. Juveniles are particularly sensitive to dietary imbalances because of their rapid growth and metabolic demand [5]. Identifying alternative protein sources that sustain performance at lower FM inclusion therefore remains a priority for the industry.

Plant- and animal-derived ingredients have been extensively evaluated as FM alternatives. Soybean meal (SBM) remains one of the most widely used plant protein source due to its availability, relatively high digestibility, and acceptable AA profile [6]. Other plant ingredients can also partially replace FM, but high inclusion levels may be constrained by AA imbalances, fiber, anti-nutritional factors, and broader sustainable trade-offs related to crop production [7,8]. Animal-derived protein ingredients, including poultry by-product meal and meat and bone meal, can complement plant ingredients and have supported high FM replacement levels in shrimp diets [9,10]. However, ingredient composition alone does not determine feed quality, as protein digestibility strongly influences nutrient utilization. In vitro digestion assays provide a rapid preliminary tool for comparing dietary protein bioaccesibility before in vivo validation [11].

Responses to FM replacement also depend on the culture system. Biofloc technology (BFT) supports intensive shrimp production by promoting microbial assimilation of nitrogenous waste and recycling nutrients within the culture system [12,13]. Biofloc biomass can provide approximately 30–50% of shrimp nutritional requirements, potentially supporting performance when dietary CP supply is reduced [14]. This supplemental trophic pathway may influence how shrimp respond to differences in dietary nutrient availability, making it important to evaluate alternative protein formulations directly under BFT conditions.

Beyond its nutritional role, BFT functions as a complex microbial ecosystem. Biofloc aggregates comprise bacteria, algae, protozoa, and organic particles derived from feed, metabolic waste, and added carbon sources [15]. These communities may influence shrimp intestinal microbiota. Associations between biofloc and intestinal bacterial communities suggest that environmental microorganisms participate in gut community assembly [16]. The intestinal microbiota of *P. vannamei* contributes to nutrient metabolism, immunity, pathogen resistance, and physiological homeostasis, and its composition is shaped by multiple factors including developmental stage, environmental conditions, farming practices, and diet [17,18]. Dietary changes can modify the abundance of specific bacterial groups [19], but the role of diet compared with temporal succession and other environmental- or host-related factors remains uncertain [20,21]. Determining whether alternative protein sources alter microbial community structure in the biofloc and shrimp intestine is therefore relevant to evaluating their broader effects under BFT.

Accordingly, the present study aimed to evaluate high fishmeal replacement using animal, plant and mixed protein sources in diets for juvenile *Penaeus vannamei* reared under biofloc technology. By addressing the dual role of diet in directly shaping gut microbiota and indirectly influencing microbial dynamics through the biofloc environment, this study provides an integrated assessment of nutritional and microbial responses to dietary strategies. Specifically, shrimp growth performance, in vitro protein digestibility, and the bacterial community structure of both biofloc and shrimp intestine were analyzed to elucidate the interactions between diet formulation, environmental microbiota, host-associated microbial communities and overall shrimp health status.

## 2. Materials and Methods

### 2.1. Ethics Approval

According to Council Directive 2010/63/EU, shrimp are not among the species requiring ethics committee approval. Nevertheless, all procedures were conducted in accordance with Annex IV of Royal Decree 53/2013, and were classified as mild. Shrimp were euthanized by cold thermal shock in an ice slurry [22]. The experiment was carried out in collaboration between the Aquaculture Laboratory of the Universitat Politècnica de València (UPV) (accreditation number ES 46 250 0001091), where animal maintenance took place, and the Universidad de Almería (UAL), where the in vitro digestibility analyses were performed.

### 2.2. Experimental Design

**Figure 1:**
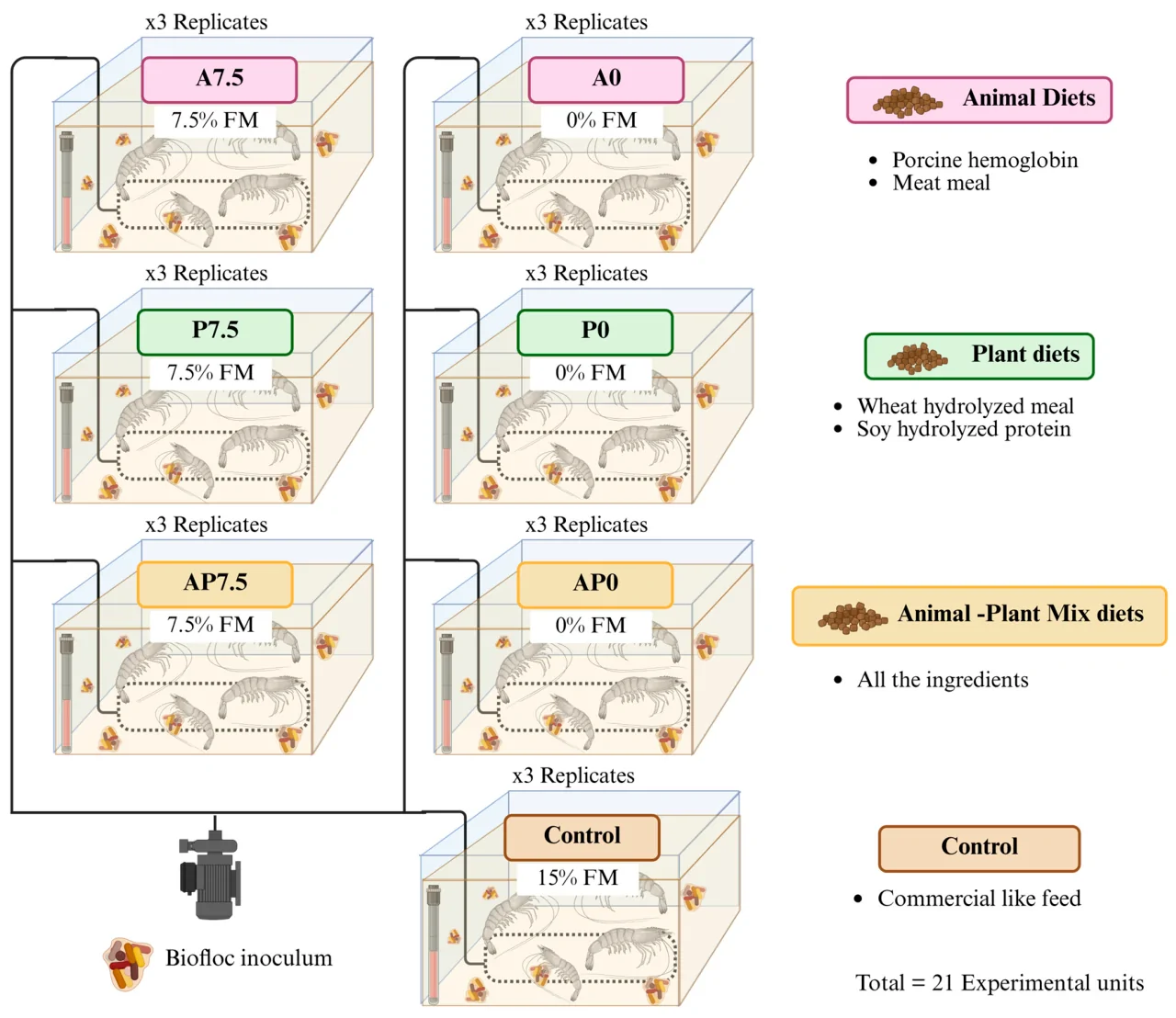
Experimental design diagram of tested feeds (A7.5, A0, P7.5, P0, AP7.5, AP0, and Control). Created in BioRender. Ferrando, S. (2026) https://BioRender.com/p0hk2p5 (accessed on 1 September 2026).

A two-factor design evaluated the effects of replacing FM with alternative protein sources on the performance of pre-juvenile *P. vannamei* reared under BFT conditions. Six experimental diets were formulated with two FM inclusion levels (0% and 7.5%), combined with three alternative protein strategies: animal- (A7.5; A0), plant-based ingredients (P7.5; P0), or a combination of both (AP7.5; AP0) (Table 1 and Figure 1). Additionally, a diet containing 15% FM was included as a practical positive control rather than as a third FM level within the factorial design. This level represents a nutritionally adequate and commercially relevant reference for juvenile *P. vannamei*, as diets containing 120–150 g/kg FM have been reported to support adequate growth performance [23,24,25]. Each dietary treatment was tested in triplicate, resulting in a total of 21 tanks. Relative to the Control, 7.5% FM represented a 50% reduction in FM ingredient inclusion, whereas 0% FM represented complete removal. Alternative ingredients were adjusted to maintain comparable target nutrient levels among formulations. In relation to animal protein sources, porcine hemoglobin and meat meal were selected as protein-rich livestock co-products with potential advantages for resource circularity, reduced dependence on marine ingredients, and formulation cost [26], while their combination provided a more balanced AA profile for FM replacement.

All diets were formulated to be isonitrogenous (38% CP) and isolipidic (10% lipid) [14]. Proximate composition was analyzed before use, and minor differences (<2%) were detected (Table 1), which were attributed to normal analytical and batch variability. Such differences are within the expected range for the manufacture of experimental aquafeeds and are unlikely to have influenced the biological responses observed. The AA profile of experimental diets was determined by quantifying total and sulfur-containing amino acids following acid hydrolysis (Table 2) [27]. Diets were produced with a semi-industrial twin screw extruder (CLEXTRAL BC-45, Firminy, St Etienne, France) following a cooking-extrusion process at 100 rpm screw speed, 110 °C temperature, and 40 atm pressure, producing pellets with a 2 mm diameter.

The economic cost of each experimental diet was estimated based on the market prices of the individual feed ingredients in December 2025, using an exchange rate of 1 USD = 0.868 €. The ingredient prices used for the calculations were as follows: fish meal, 1.39 €/kg; fish oil, 2.00 €/kg [28]; wheat meal, 0.05 €/kg; soybean meal, 0.17 €/kg [29]; porcine hemoglobin, 1.32 €/kg; wheat hydrolyzed meal, 2.40 €/kg; soy hydrolyzed meal, 1.16 €/kg; potato protein, 1.70 €/kg; Lithonutri seaweed, 0.65 €/kg [30]; meat meal, 0.79 €/kg [31]; soybean oil, 0.95 €/kg [32]; calcium phosphate, 0.91 €/kg [33]; taurine, 2.15 €/kg [34]; vitamin premix, 74.99 €/kg [35]; and soy lecithin, 0.95 €/kg [36]. These prices were used to estimate the cost of each diet (Table 1).

**Table 1:** Formulation and proximate composition of the juvenile stage experimental diets (g/kg dry basis).

	A7.5	A0	P7.5	P0	AP7.5	AP0	Control
**Feed** **ingredients (g/kg dry basis)**							
Fishmeal	75	0	75	0	75	0	150
Wheat meal	405	382	333	324	358	322	334
Soy meal	0	0	215	218	163	188	210
Porcine hemoglobin	100	120	0	0	20	37	0
Meat meal	275	340	0	0	80	110	0
Wheat hydrolyzed meal	0	0	70	90	55	60	50
Soy hydrolyzed protein	0	0	70	90	55	60	50
Potato protein	0	0	70	90	55	60	50
Lithonutri seaweed	50	50	50	50	50	50	50
Fish oil	28	36	28	36	28	36	20
Soy oil	32	22	39	32	26	27	46
Pre-mixed vitamins	10	10	10	10	10	10	10
Calcium phosphate	15	25	20	25	15	25	15
Lecithin soy	10	15	10	15	10	15	5
Taurine ^1^	0	0	10	20	0	0	0
**Proximate composition (%)**							
Crude protein (CP)	38.4	37.9	39.7	39.2	38.7	38.7	39.2
Crude lipids (CL)	9.6	9.5	9.3	8.8	9.4	9.6	9.9
Carbohydrates ^2^	36.7	41.0	34.1	40.0	42.1	42.2	40.2
Ash	15.3	11.6	16.9	12.0	9.8	9.5	10.7
**€**/kg^2^	1.36	1.36	1.45	1.49	1.41	1.42	1.41

^1^ Taurine was supplemented only in plant-based diets to compensate for the amino acid profile of the plant protein ingredients and to achieve a more balanced dietary amino acid composition [37]. ^2^ Carbohydrates were calculated as 100 − (CP + CL + Ash).

**Table 2:** Amino acid profiles of the experimental diets (A7.5, A0, P7.5, P0, AP7.5, AP0, and Control), including essential and non-essential amino acid compositions (g/100 g protein).

	A7.5	A0	P7.5	P0	AP7.5	AP0	Control
**Essential** **AA (EAA)**							
Arginine (ARG)	5.81	5.91	5.88	5.85	5.90	5.85	5.80
Histidine (HIS)	3.22	3.14	2.24	2.12	2.39	2.48	2.28
Isoleucine (ILE)	3.05	2.70	4.56	4.44	3.94	3.81	4.73
Leucine (LEU)	8.91	8.69	8.25	8.17	8.33	8.55	8.33
Lysine (LYS)	5.88	5.68	5.09	4.99	5.22	5.04	5.50
Methionine (MET)	1.03	0.86	1.39	1.31	1.29	1.24	1.21
Phenylalanine (PHE)	5.04	4.89	5.31	5.44	5.12	5.29	5.26
Threonine (THR)	3.80	3.55	4.25	4.12	4.02	3.90	4.31
Valine (VAL)	6.14	5.98	5.22	5.10	5.38	5.51	5.27
**Non-essential AA (NEAA)**							
Alanine (ALA)	6.78	7.29	4.52	4.19	5.48	5.39	4.83
Aspartic acid (ASP)	9.41	9.29	9.72	9.75	9.54	9.63	9.78
Cysteine (CYS)	0.65	0.55	1.02	0.99	0.91	0.95	0.82
Glutamic acid (GLU)	17.03	16.36	21.94	23.02	20.30	20.34	21.54
Glycine (GLY)	8.44	10.11	4.77	4.40	6.65	6.45	5.02
Proline (PRO)	7.35	7.98	6.91	7.18	7.29	7.37	6.73
Serine (SER)	4.86	4.71	5.31	5.41	5.11	5.17	5.30
Tyrosine (TYR)	2.61	2.31	3.62	3.52	3.15	3.03	3.27
*EAA*	42.86	41.41	42.19	41.54	41.57	41.67	42.71
*NEAA*	57.14	58.59	57.81	58.46	58.43	58.33	57.29
*EAA/NEAA*	0.75	0.71	0.73	0.71	0.71	0.71	0.75
*EAA/Total AA*	0.43	0.41	0.42	0.42	0.42	0.42	0.43

*P. vannamei* post-larvae (PL12) were sourced from White Panther Company (Austria). Post-larvae were gradually acclimated to 21 g/L salinity and fed a commercial diet (LeGoussant–Vanna PL) until reaching 2.55 ± 0.04 g, when they were randomly distributed into 1 m^3^ polyethylene tanks at 350 shrimp/m^3^. Each tank was equipped with continuous aeration via a venturi system, an independent heating unit, and Needlone strips to provide additional surface area for biofilm development. A natural light–dark cycle was maintained throughout the experiment.

### 2.3. In Vitro Digestibility Assays

Potential differences in protein bioaccessibility among ingredients were preliminarily assessed using an in vitro digestion assay developed for this study, which was subsequently applied to the formulated diets. The enzyme extract was prepared from the digestive glands of 30 *P. vannamei* individuals with a mean body weight of approximately 10 g. Enzyme donors were selected independently from the shrimp used in the growth trial to avoid potential treatment-related differences in digestive enzyme secretion. During preliminary method optimization, extracts from smaller (~2 g) and larger (~10 g) shrimp produced comparable relative protein-hydrolysis patterns under the standardized assay conditions (unpublished data). Larger shrimp were therefore selected for methodological reasons, because they provided sufficient hepatopancreatic tissue to prepare a single homogeneous pooled enzyme batch for all ingredients, diets, and replicates, minimizing analytical variability. The hepatopancreas samples were homogenized in distilled water at a ratio of 1:3 (*w*:*v*) and centrifuged at 12,000× *g* for 15 min at 4 °C. The resulting supernatant was aliquoted and stored at −20 °C until analysis. Trypsin activity was determined using 50 mM Nα-Benzoyl-DL-arginine p-nitroanilide (BAPNA) as substrate [38].

Assays were performed at pH 8.0, based on the reported optimum for *P. vannamei* trypsin [39], with 60 mM Na^+^ and 24 mM Ca^2+^, concentrations that produced maximum trypsin activity during preliminary optimization (unpublished data). A physiologically based enzyme ratio of 0.038 mU trypsin/mg was established from the total trypsin activity of the enzyme donors and their estimated daily protein intake. Each assay contained 420 mg of ingredient or 417 mg of formulated feed, corresponding approximately to the ration of a 10 g shrimp fed at 8% body weight with a 35% CP diet. Digestion was conducted for 5 h at 28 °C. Semi-permeable membrane bioreactors were used, adapted from [40]. Each bioreactor consisted of two chambers separated by a 3.5 kDa MWCO membrane (ZelluTrans, Carl Roth GmbH, Karlsruhe, Germany). Digestion was initiated after adjustment to pH 8.0 with 0.1 M borate buffer by adding the enzyme extract. Hydrolysis products permeating into the lower chamber were collected at 1, 2, 3.5, and 5 h under a continuous flow of the same buffer, and released amino groups were quantified using ortho-phthalaldehyde [41]. Bioreactors were maintained at 28 °C throughout the assay. All assays were performed in triplicate. A blank containing heat-inactivated enzyme extract was included for each ingredient to account for basal amino acid release unrelated to enzymatic hydrolysis. Results were expressed as AA released (mg/100 mg feed), estimated protein bioaccessibility (%), and cumulative product release over time.

### 2.4. Shrimp Growth and Feed Utilization

Shrimp were manually fed three times per day. The scheduled ration was based on the estimated shrimp biomass and a customized feeding table based on previous studies [14,42]. Feeding trays were visually inspected before each meal for uneaten feed. When uneaten feed was detected, subsequent ration was reduced by 10%; this adjustment was repeated if feed remained at the next inspection, whereas the scheduled ration was restored when no uneaten feed was observed. Feeding rates were adjusted weekly according to the biomass estimated from the growth samplings, using the same procedure in all tanks. Because uneaten feed was not quantitatively recovered, all feed use indicators were calculated from feed supplied rather than actual feed intake and are therefore reported as apparent estimates. Supplied feed not consumed by shrimp could have remained uneaten or been incorporated into the biofloc and utilized by microorganisms.

Shrimp growth was monitored weekly by randomly sampling and weighing 15% of the population in each tank. Sampling was conducted at 09:00 h, before the first daily feeding under identical conditions for all treatments. Final body weight was recorded on day 74. Growth and feed use indicators were calculated as follows:

[1] Specific growth rate (SGR, %/day) = 100 × (ln _final_ _weight_ − ln _initial weight_)/days;

[2] Feed conversion ratio (FCR) = feed supplied (g)/biomass gain (g);

[3] Feed intake (FI, g/100 g shrimp · day) = 100 × feed supplied (g)/average biomass (g) × days;

[4] Protein intake ratio (PIR) = FI × dietary crude protein (%);

[5] Protein efficiency ratio (PER) = Shrimp weight gain (g)/total protein supplied (g);

[6] Survival (%) = Final nº of shrimp × 100/initial nº of shrimp.

Economic feed efficiency was assessed using the economic conversion ratio (ECR) [43]:

[7] ECR (€/kg shrimp biomass produced) = FCR × price of the diet (€/kg).

### 2.5. Shrimp and Biofloc Sampling

Shrimp and biofloc samples were randomly collected at the beginning and end of the experiment. Whole shrimp intended for nutritional composition analysis was frozen at −20 °C and/or lyophilized. For microbiome analysis, intestines were dissected, flash-frozen in liquid nitrogen, and stored at −80 °C. Biofloc samples for nutritional composition were obtained by settling culture water for 1 h, collecting the floc layer, and drying it at 60 °C for 24 h. Fresh biofloc for microbiome analysis was collected in sterile 50 mL tubes and stored at −80 °C.

### 2.6. Nutritional Composition of Shrimp and Biofloc

Dry matter (DM) and ash were determined according to method 934.01 and 942.05, by drying at 105 °C until constant weight and incineration at 550 °C for 5 h, respectively [44]. Nitrogen (N) and carbon (C) were determined using a Leco CN628 Elemental Analyzer (Leco Corporation, St. Joseph, MI, USA), following method 920.39. Gross energy (GE) was calculated as GE (MJ/Kg) = 51.8 × C − 19.4 × N [45]. Organic matter (OM, %) was calculated as the difference between biofloc dry matter and ash content.

### 2.7. 16S rRNA Gene Analysis

DNA for 16S rRNA was extracted from biofloc using the NucleoSpin^®^ eDNA Water (Macherey-Nagel, Düren, Germany) by direct precipitation without filtration and from shrimp intestine using the QIAamp PowerFecal Pro DNA Kit (Qiagen, Hilden, Germany). Sequencing was carried out using the Sequel II Sequencing Kit 2.0 (PacBio, Menlo Park, CA, USA) on the Sequel II PacBio system. Full-length 16S rRNA genes were amplified with universal primers 27F: AGGRGTTYGATYMTGGCTCAG and 1492R: RGYTACCTTGTTACGACTT, both containing sample-specific PacBio barcodes for multiplexing. PCR amplification was performed with KAPA HiFi HotStart DNA Polymerase (KAPA Biosystems, Wilmington, MA, USA) following the PacBio protocol for full-length 16S rRNA gene amplification with barcoded primers (Part No. 101-599-700, Version 04). Amplification consisted of 27 cycles of denaturation at 95 °C for 30 s, annealing at 57 °C for 30 s, and extension at 72 °C for 60 s. Amplicons were quality-checked using a Fragment Analyzer (Agilent Technologies, Santa Clara, CA, USA) and pooled at equal concentrations. Libraries were prepared using the SMRTbell Express Template Prep Kit 2.0 (PacBio, Menlo Park, CA, USA) following the same PacBio protocol. Library concentration and size were assessed using the Qubit HS DNA kit (Qubit Fluorometer, Invitrogen, Carlsbad, CA, USA) and Fragment Analyzer (Agilent Technologies, Santa Clara, CA, USA), respectively. Primer annealing and polymerase binding were performed with the Sequel II Binding Kit 2.1 and Sequel II DNA Internal Control Complex 1.0 (PacBio, Menlo Park, CA, USA). Sequencing was conducted on a PacBio Sequel II system using the Sequel II Sequencing Kit 2.0.

Raw FASTQ reads were processed in QIIME 2 (version 2021.4) [46]. Denoising and chimera removal were performed using DADA2 [47]. Sequences were aligned with MAFFT [48], and phylogenetic trees were constructed using FastTree2 [49]. Taxonomic classification was performed with the QIIME 2 Naive Bayesian classifier [50] against the SILVA 138 database [51]. Taxon proportions were calculated from amplicon sequence variants (ASVs) and transformed using the centered log-ratio (CLR), with a pseudocount of 0.5 assigned to zero observations, using the “transformCount” function of the R package “mia” [52].

### 2.8. Water Quality Analysis

Water quality was monitored throughout the experiment. Temperature, dissolved oxygen (DO) and salinity were measured daily using HANNA multiparameter equipment (HI19829, Woonsocket, RI, USA) and YSI ProODO (Yellow Springs, OH, USA). Weekly monitoring included pH (pH50, XS Instruments, Capri, Italy), alkalinity (HANNA Checker HI755, Woonsocket, RI, USA), ammonium (NH_4_^+^), nitrite (NO_2_^−^), and total suspended solids (TSS). pH and alkalinity were adjusted when necessary by adding NaHCO_3_. TSS was quantified using Whatman glass fiber filters (0.45 µm), and excess sediment was decanted when TSS exceeded 500 mg/L [53]. Ammonium (NH_4_^+^, mg/L) and nitrite (NO_2_^−^, mg/L) concentrations were determined using standard photometric methods [54,55,56]. Nitrate (NO_3_^−^, mg/L) and phosphate (PO_4_^3−^, mg/L) concentrations were measured at the beginning and end of the experiment using photometric methods [55,57,58].

Initial water conditions were 28 °C, 20 g/L salinity, 150–300 mg/L alkalinity, pH 7.5–8.5, and DO > 5 mg/L. Mature biofloc was established in tanks using an inoculum of 125 mg/L decanted biofloc over pre-chlorinated seawater (5% sodium hypochlorite). No water exchanges were performed during the experiment, with only freshwater added to compensate for evaporation losses [59].

### 2.9. Statistical Analysis

Statistical analyses for in vitro digestibility analysis, shrimp growth performance, nutritional composition and environmental parameters were conducted using the Statgraphics Centurion XVI software package ( version 16) (StatPoint Technologies, Warrenton, VA, USA). Normality was assessed using skewness and kurtosis values, normal probability plots, and the Shapiro–Wilk test. When the assumptions were met, one-way ANOVA was performed, followed by the Newman–Keuls post hoc test. When data did not meet the assumptions of normality, the non-parametric Kruskal–Wallis test was used to assess overall differences among treatments, and Bonferroni-adjusted post hoc tests were applied to determine significant pairwise differences. The microbiome taxonomic profiling statistical analyses were performed in R software (version 4.2.0; R Core Team, Vienna, Austria) using open-source packages including Biostrings, vegan, and ALDEx2. Given the compositional nature and expected non-normal distribution of microbiome data, a non-parametric framework was adopted. Differential abundance analyses were conducted using the Wilcoxon test as implemented in ALDEx2 (version 1.28.1), and *p*-values were adjusted for multiple testing using the Benjamini–Hochberg procedure. Statistical significance was set at *p* < 0.05, and results are presented as means ± standard error (SE).

## 3. Results

### 3.1. Effect of Diet on Water Quality

Water quality remained within ranges suitable for *P. vannamei* culture throughout the trial (Table 3). Significant differences among dietary treatments were detected for dissolved oxygen (DO), salinity, temperature, and alkalinity (Supplementary Material Table S1); however, these differences were small and showed no consistent diet-related pattern.

### 3.2. In Vitro Digestibility Feed Assay

Dietary treatment significantly affected both final AA release and estimated protein bioaccessibility (Table 4), indicating that the origin and processing of the dietary protein sources influenced their susceptibility to enzymatic hydrolysis. The P0 diet exhibited significantly higher values than all other treatments, likely due to its greater inclusion of hydrolyzed plant protein ingredients, which may have increased peptide bond accessibility to digestive enzymes. In contrast, the P7.5 and Control diets showed intermediate values. Diets based on animal-derived protein sources exhibited the lowest protein bioaccessibility, suggesting reduced in vitro digestibility of ingredients such as porcine hemoglobin and meat meal. Mixed diets displayed intermediate values, consistent with the combined contribution of plant- and animal-derived protein sources. Supplementary Material Figure S1 illustrates the amount of AA released over time (1, 2, 3.5, and 5 h), illustrating the progression of the in vitro digestion of the protein hydrolysis in the tested diets.

### 3.3. Shrimp Growth Performance

The tested diets significantly affected feed utilization parameters in *P. vannamei*, specifically the apparent FCR and PER (Table 5). Shrimp fed the A7.5 and AP7.5 diets exhibited significantly lower FCR values than those fed AP0, indicating a more efficient apparent conversion of the supplied feed into shrimp biomass. Similarly, diet A7.5 resulted in the highest apparent PER, with significant differences in comparison with AP0, which showed the lowest protein efficiency. However, these differences in feed utilization were not reflected in protein or energy retention efficiencies, which remained comparable among treatments. Likewise, no significant effects of dietary treatment were observed on final body weight, SGR, FI, PIR, or survival, although some numerical variation was detected among treatments.

### 3.4. Shrimp and Biofloc Nutritional Composition

The initial shrimp exhibited significantly higher GE content but lower CP content than shrimp sampled at the end of the trial (Table 6). This temporal pattern indicates a shift toward a greater relative contribution of body protein and a lower energy density as a shrimp grows. However, no significant differences in shrimp proximate composition were detected among dietary treatments, indicating that the replacement of FM and the use of different alternative protein sources did not substantially alter whole body nutrient deposition. Regarding biofloc composition, organic matter (OM) and CP contents generally increased throughout the trial, except in the animal diets, where they decreased. Similarly, biofloc associated with plant diets exhibited higher energy values than that associated with animal diets, while mixed diets showed intermediate values, with no significant differences relative to the other treatments. In contrast, ash content decreased in plant-based diets throughout the experiment period, indicating that dietary composition influenced mineral accumulation in the biofloc.

### 3.5. Diet Economic Analysis

**Figure 2:**
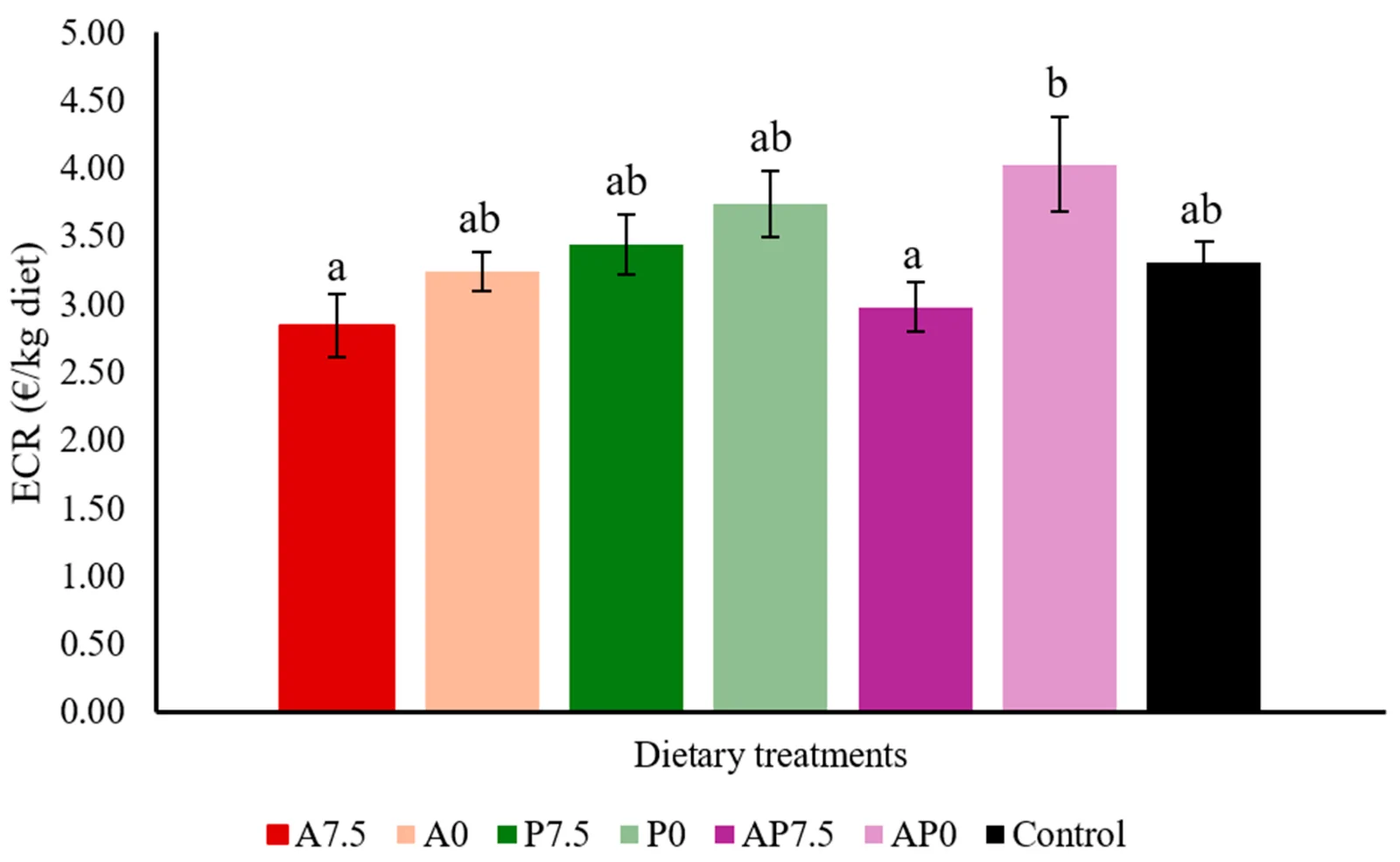
Economic conversion ratio (ERC, €/kg diet) under the dietary treatments tested (A7.5, A0, P7.5, P0, AP7.5, P0 and Control) (mean ± SE, *n* = 3). Different letters indicate differences among dietary treatments (*p* < 0.05). Newman-Keuls post hoc test.

The apparent ECR was significantly affected by dietary treatment (Figure 2). The A7.5 and AP7.5 diets showed the lowest ECR values, differing significantly from AP0, which exhibited the highest value. In practical terms, feeding shrimp with A7.5 or AP7.5 reduced feed costs by approximately 1.12 € per kilogram of shrimp biomass produced, compared with AP0. This greater apparent cost-efficiency was consistent with the lower apparent FCR observed for these diets, indicating that the inclusion of 7.5% FM together with animal-derived alternative protein sources provides a more favorable balance between feed cost and biomass production. Conversely, the higher ECR of AP0 suggests that the lower cost of completely replacing FM did not compensate for its poorer apparent feed conversion.

### 3.6. Shrimp Intestine and Biofloc Microbiome Characterization

To assess the effect of different feed ingredients on microbiome composition, 16S rRNA gene sequencing was performed on biofloc and shrimp intestinal samples. A total of 1,405,114 and 3,179,712 sequencing reads were generated from the biofloc and intestinal samples, respectively. Across all treatments, at least 81% of the reads were non-chimeric, indicating good sequencing quality (Supplementary Material Table S2). To aid interpretation, results were summarized according to temporal stage (Initial and Final), ingredient base (Animal, Plant, Mixed), fishmeal inclusion level (0 and 7.5%), and individual dietary treatments (A7.5, A0, P7.5, P0, AP7.5, AP0, and Control).

#### 3.6.1. α & β Diversity

For α-diversity, Chao1, Simpson, and Shannon indices were calculated to provide a comprehensive assessment of species richness and evenness (Table 7). Significant differences were detected between the initial and final sampling points in both the biofloc and shrimp samples, although opposite trends were observed. In the biofloc, diversity indices were higher during the immature stage and decreased as the system matured. In contrast, intestinal microbiota diversity increased over time, likely reflecting the progression of shrimp growth and microbiome development.

**Figure 3:**
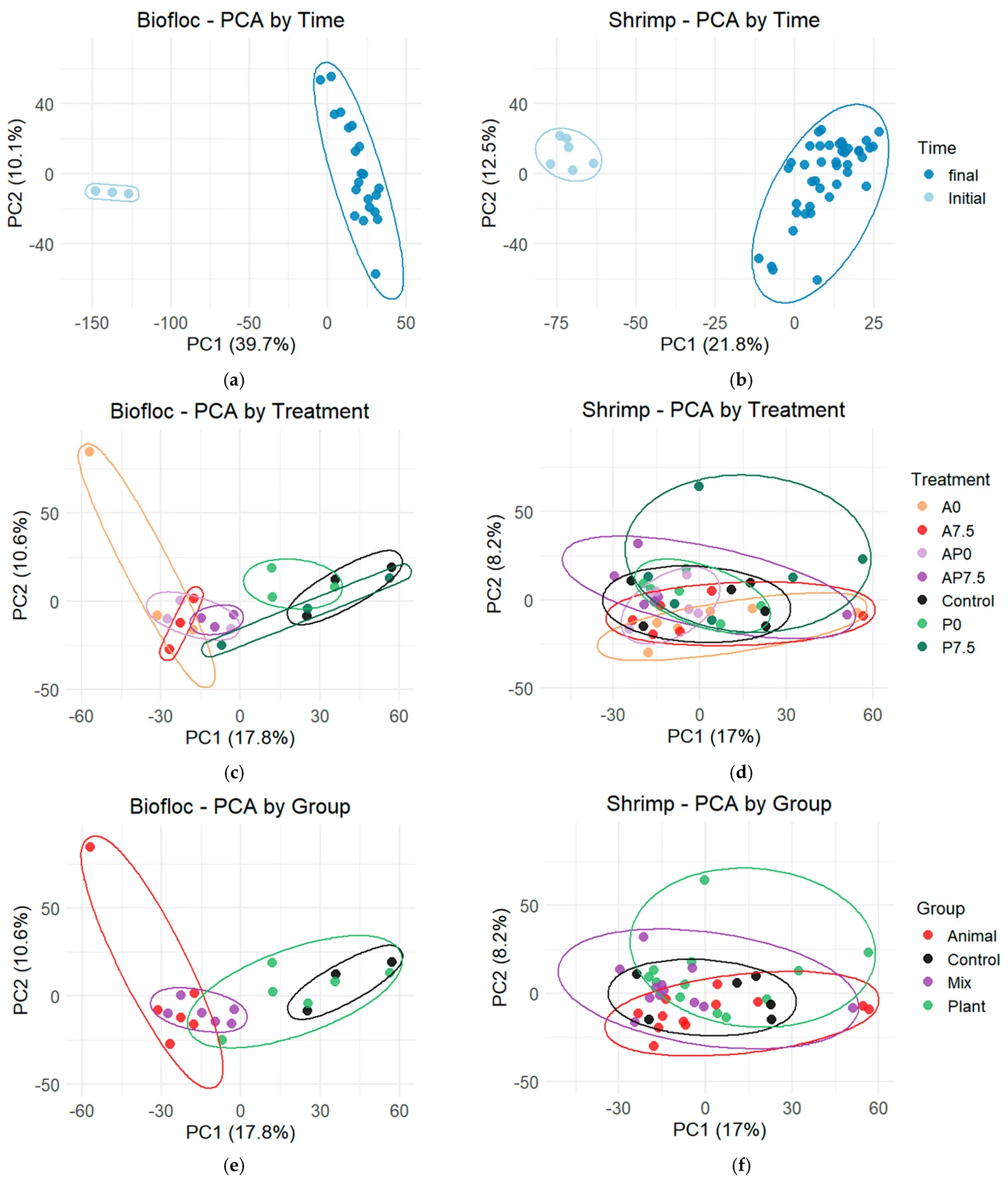
Principal component analysis (PCA) of microbial beta-diversity in biofloc (**a**,**c**,**e**) and shrimp intestine samples (**b**,**d**,**f**). Panels show comparisons by sampling time (**a**,**b**), dietary treatments (**c**,**d**), and dietary treatments grouped according to primary ingredient source (**e**,**f**) (*n* = 3).

β diversity analyses revealed significant temporal changes in community structure in both biofloc and shrimp intestine (Figure 3; Table 8). Time explained 38.6% of the variation in biofloc and 20.4% in intestinal communities. PERMDISP also detected significant differences in dispersion, indicating that temporal variation reflected not only shifts in community composition but also changes within-group variability. These results suggest that both communities underwent progressive ecological succession throughout the experimental period, although they followed distinct trajectories.

Dietary treatment also affected community composition in both biofloc and shrimp, although the response patterns differed between them. In the biofloc, non-pairwise comparisons remained significant after multiple testing correction, suggesting that dietary effects were broadly distributed across treatments. In contrast, intestinal AP0 communities differed from A0, A7.5, P0, and P7.5 (Figure 3c,d). These findings indicate that dietary effects were more specifically reflected in the intestinal microbiota than in the surrounding biofloc community.

Ingredient source significantly influenced both communities. In the biofloc, animal-based diets differed significantly from both plant and mixed diets, whereas the Control differed only from the mixed diets. In contrast, the shrimp intestinal microbiota showed significant differences between plant-based diets and both animal and mixed diets, while the Control did not differ significantly from any of the other dietary groups (Figure 3e,f).

Finally, the FM inclusion level had a comparatively limited effect: an overall difference was detected in biofloc, although no pairwise comparison remained significant after correction, whereas no effect was detected in the intestine. Collectively, these results indicate that the origin of the dietary ingredients was a stronger driver of microbial community differentiation than the level of fishmeal inclusion within the range evaluated.

#### 3.6.2. Microbiota Taxonomic Profiles

Across biofloc and shrimp, a total of 29 phyla, 158 orders, and 240 families were identified. Taxa with less than 1% relative abundance and unclassified taxa were grouped under the category “Others.” This filtering resulted in 10 phyla, 27 orders, and 28 families for visualization.

**Figure 4:**
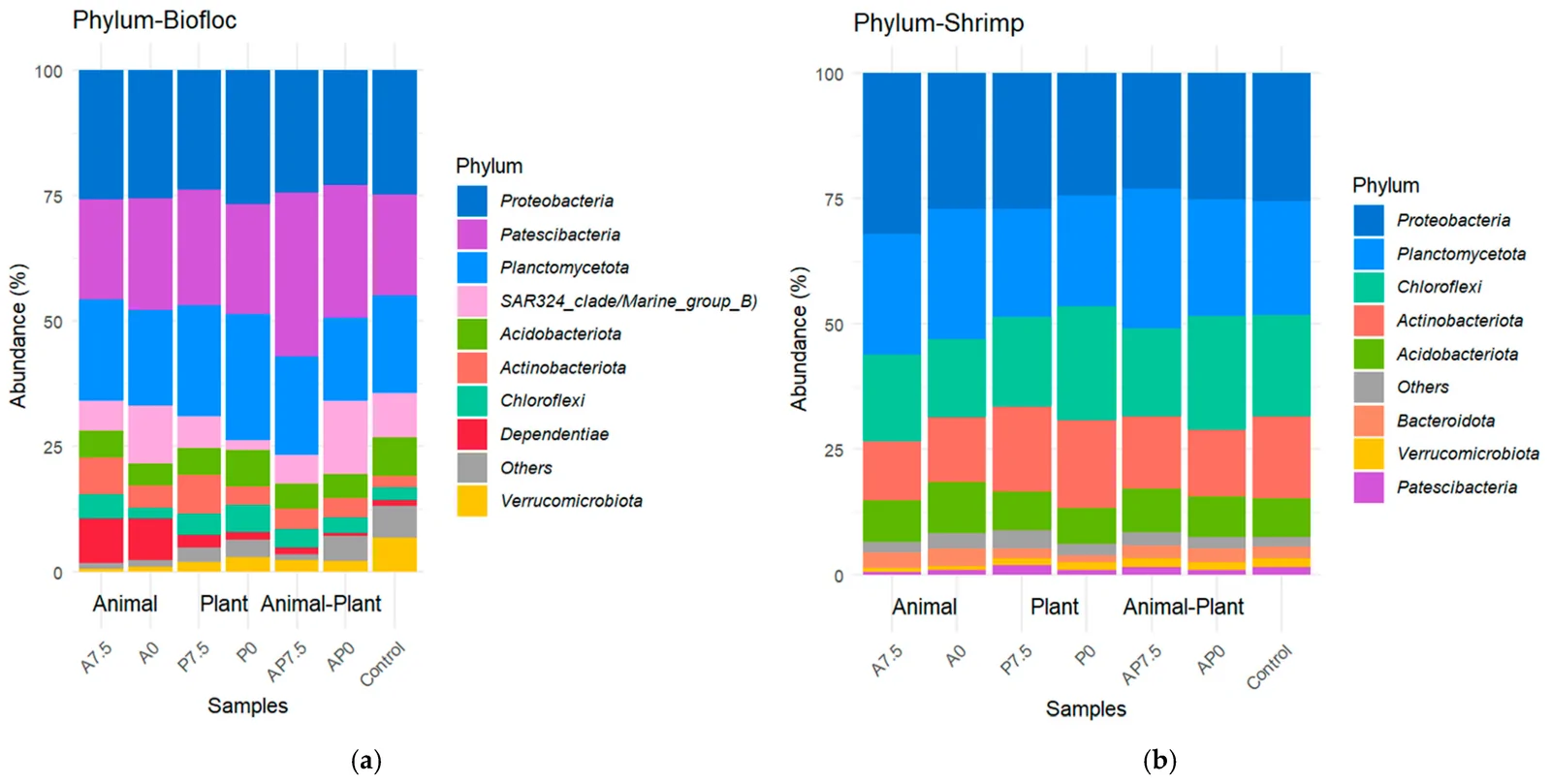

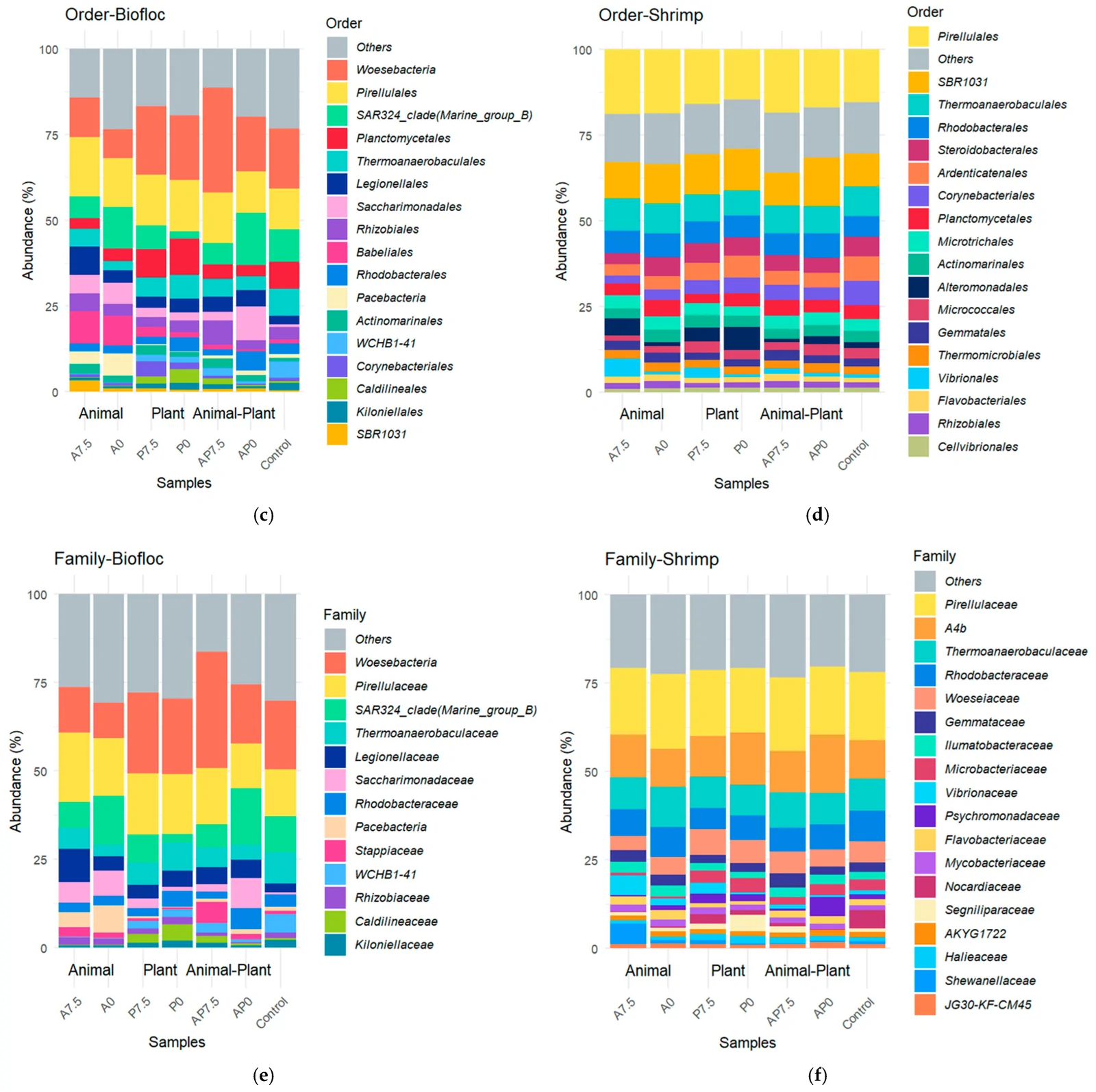
Relative abundance (%) of microbiome composition at (**a**,**b**) the phylum, (**c**,**d**) order and (**e**,**f**) family levels for biofloc and shrimp intestine samples at the end of the experiment for the tested diets.

At the end of the experiment, the most abundant phyla (>10%) in biofloc were *Proteobacteria* (24.84%), *Patescibacteria* (23.85%), and *Planctomycetota* (20.28%), together accounting for 69% of the relative abundance (Figure 4a). *Woesebacteria* (16.53%) and *Pirellulales* (13.48%) were the dominant orders, while *Woesebacteria* (14%) and *Pirellulaceae* (12%) were the most abundant families (Figure 4b,c). In shrimp intestine, *Proteobacteria* (26.42%), *Planctomycetota* (24.86%), *Chloroflexi* (19.12%), and *Actinobacteriota* (14.75%) together represented 84% of the community (Figure 4d). *Pirellulales* (16.68%) and *SBR1031* (11.20%) were the dominant orders, while *Pirellulaceae* (18%) was the dominant family (Figure 4e,f).

**Figure 5:**
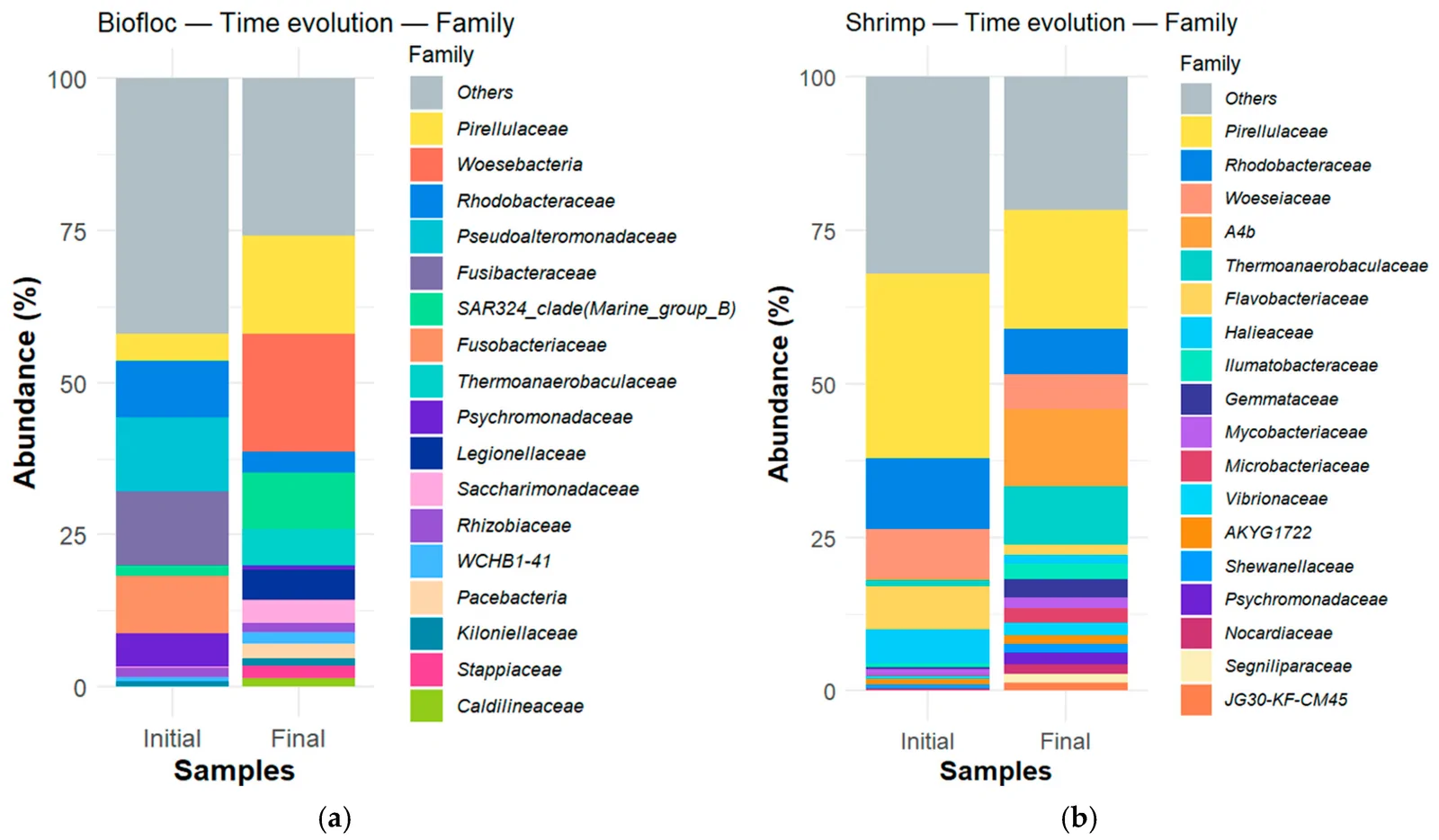
Time evolution of microbial family composition in (**a**) biofloc and (**b**) shrimp intestine samples.

Family-level composition was further compared according to sampling time, dietary ingredient source, and individual dietary treatment. Marked temporal changes occurred in both microbial communities, although their taxonomic trajectories differed. Only a limited number of families showed a shared temporal response across compartments: *Rhodobacteraceae* was more abundant initially, whereas *Thermoanaerobaculaceae* increased at the final sampling in both biofloc and intestine. Beyond these common patterns, temporal changes were predominantly compartment-specific. Initial biofloc communities showed higher abundances of *Fusibacteraceae*, *Fusobacteriaceae*, *Pseudoalteromonadaceae*, and *Psychromonadaceae*, whereas the mature biofloc was enriched in *Caldilineaceae*, *Kiloniellaceae*, *Legionellaceae*, *Pacebacteria*, *Pirellulaceae*, *Rhizobiaceae*, *Saccharimonadaceae*, SAR324 clade, *Stappiaceae*, *WCHB1-41*, and *Woesebacteria*. In the shrimp intestine, *Flavobacteriaceae*, *Halieaceae*, *Pirellulaceae*, and *Woeseiaceae* were more abundant initially, whereas *A4b*, *Gemmataceae*, *Ilumatobacteraceae*, *Microbacteriaceae*, and *Segniliparaceae* increased at the final sampling point (Figure 5a,b; Supplementary Tables S3 and S4). These results indicate that temporal development affected both biofloc and shrimp gut, but bacterial families were selected differently, consistent with distinct ecological conditions in the biofloc matrix and the intestinal environment. Additionally, *Psychromonadaceae*, *Shewanellaceae*, and *Vibrionaceae* stability suggested greater temporal persistence of these families within the intestinal community regardless of the tested diet.

**Figure 6:**
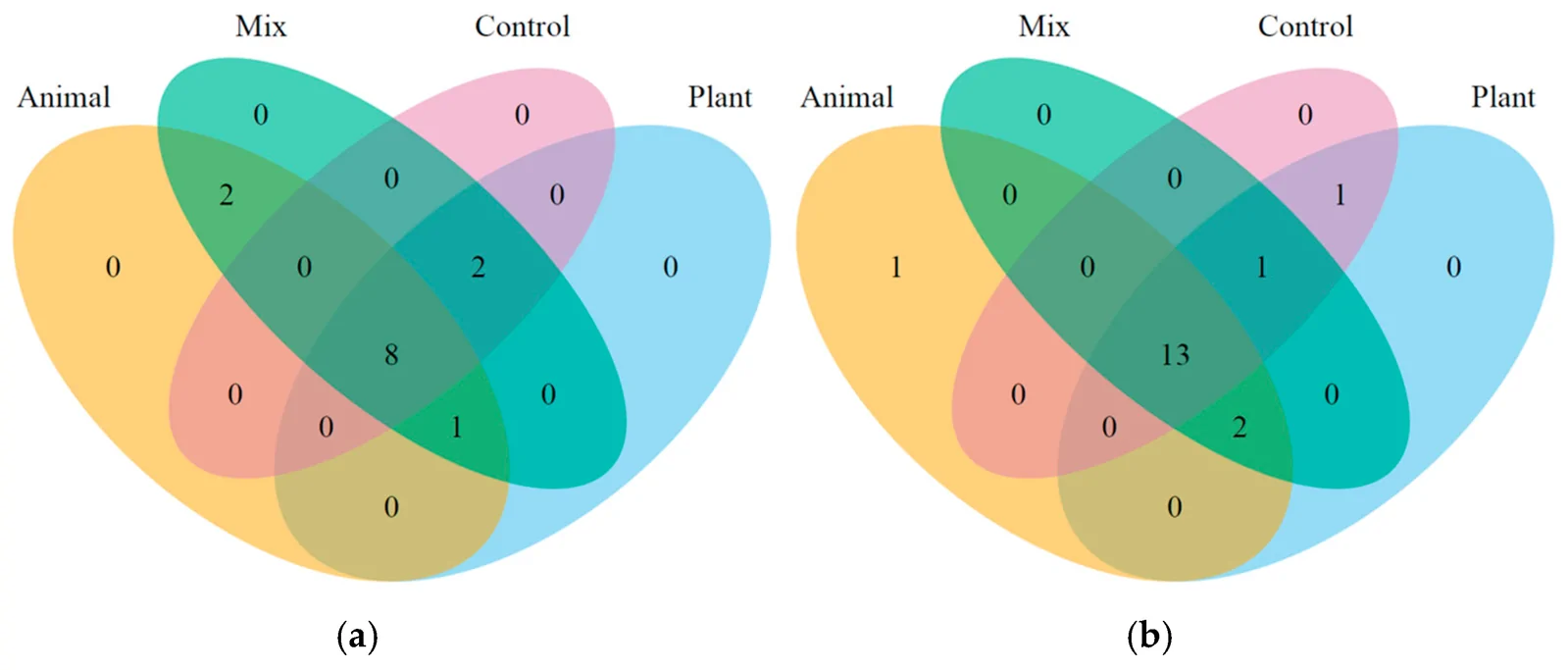
The Venn diagram shows the comparison of taxonomic groups (family level) with more than 1% abundance between (**a**) biofloc and (**b**) shrimp intestine samples at the end of the juvenile phase.

Dietary ingredient source affected a limited and mostly compartment-specific subset of families (Figure 6, Supplementary Material Tables S5–S7). Compared with animal-based diets, plant-based diets showed higher abundances of *WCHB1-41* and *Kiloniellaceae* in biofloc and *Halieaceae* and *Microbacteriaceae* in the intestine. In biofloc, animal-based diets also showed higher levels of *Saccharimonadaceae*, *Rhizobiaceae*, and *Pacebacteria* than in the Control, Mixed, and Plant groups, respectively; meanwhile, in the intestine, *Flavobacteriaceae* and *Shewanellaceae* were more abundant than with plant-based diets. Several dominant families, including *Pirellulaceae* and *Woesebacteria* did not differ significantly among ingredient source groups, suggesting that the microbial community remained relatively stable across dietary protein source categories, although plant- and animal-derived ingredients selectively affected specific bacterial families.

Differences among individual diets were fewer and generally consistent with the ingredient-source analysis. In biofloc, *WCHB1-41* and *Kiloniellaceae* were significantly more abundant in P0 than in A7.5, consistent with their enrichment in the Plant group relative to the Animal group. *Rhizobiaceae* showed higher abundance in A7.5 than in AP treatments, in agreement with its higher abundance in the Animal group relative to the Mixed group (Supplementary Material Table S8). For the shrimp intestine samples, significant treatment-related differences were restricted to *Microbacteriaceae.* This family was more abundant in P7.5, P0, and AP0 and less abundant in the animal-based diets, particularly A0, whereas the Control and AP7.5 showed intermediate and more variable values (Supplementary Material Table S9).

**Figure 7:**
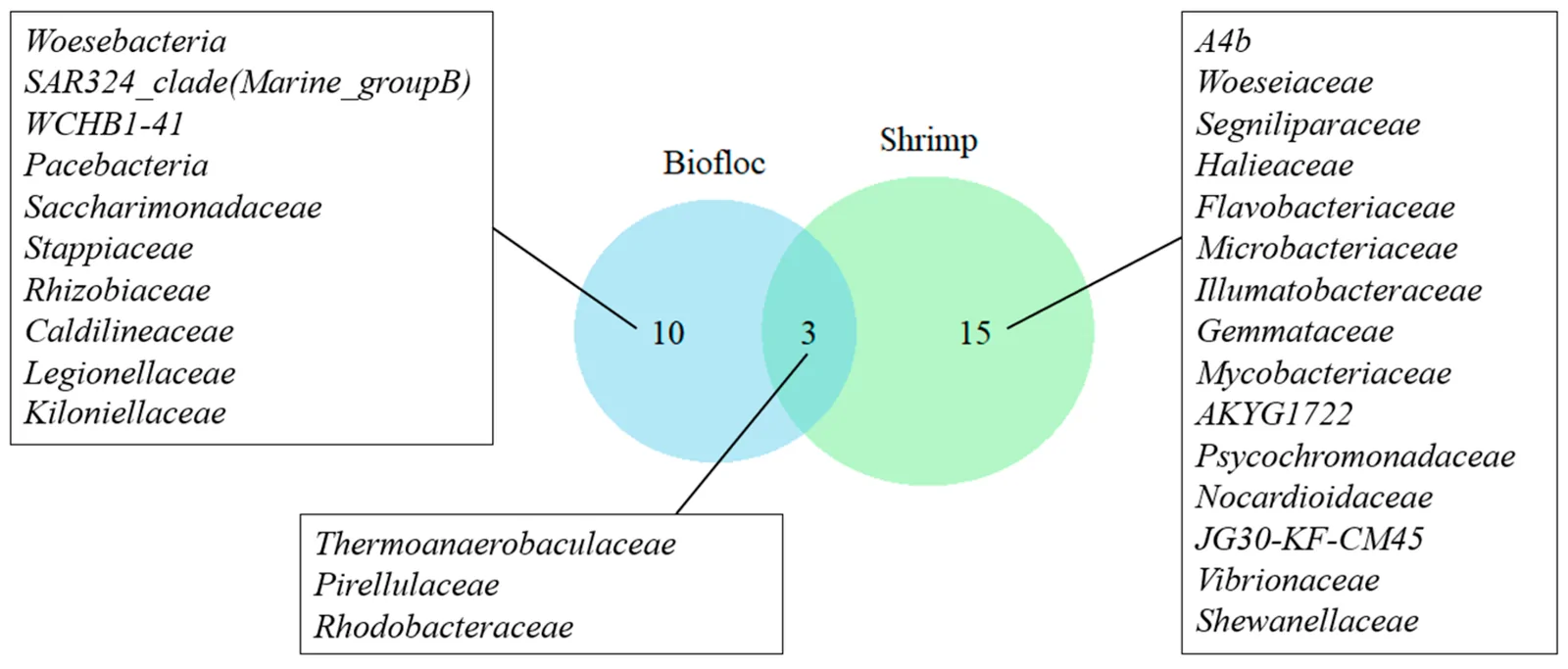
The Venn diagram shows the comparison of taxonomic groups (family level) between biofloc and shrimp intestine samples at the end of the juvenile phase.

Direct comparison of microbial communities in biofloc and shrimp intestine samples further revealed a limited family-level overlap. *Thermoanaerobaculaceae*, *Pirellulaceae*, and *Rhodobacteraceae* were shared between the two compartments, whereas most families were compartment-specific. The complete distribution of shared and compartment-specific families is shown in Figure 7. Collectively, the coexistence of a small shared microbial core with a larger proportion of compartment-specific families indicates that, despite continuous interaction between shrimp and the surrounding biofloc, each habitat exerted distinct selective pressures on microbial establishment.

## 4. Discussion

The present study demonstrates that the response of *P. vannamei* to fishmeal replacement under BFT conditions cannot be predicted solely from the digestibility of the formulated diets. Although plant-based diets showed the highest protein bioaccessibility in vitro, these differences were not reflected in shrimp growth. Instead, diets containing 7.5% fishmeal supplemented with animal or a combination of animal- and plant-derived protein sources provided the most favorable apparent feed utilization and economic responses while maintaining growth comparable to the Control. Dietary formulation also modified biofloc nutritional composition and specific bacterial taxa. However, temporal succession associated with biofloc maturation and shrimp development exerted the strongest influence on overall microbial community structure.

In vitro digestion assays are useful preliminary screening tools because they provide a rapid comparative assessment of protein bioaccessibility prior to in vivo validation [60]. In the present study, plant-based diets exhibited the highest AA release and protein bioaccessibility. These results suggest greater susceptibility of the plant protein fraction to proteolytic hydrolysis and are consistent with ingredient-level digestibility assessments [61,62]. The inclusion of hydrolyzed soy and wheat proteins may have further facilitated peptide bond cleavage and AA release [63]. Conversely, the lower values obtained for the animal-based diets are consistent with previous studies showing that blood and meat-derived ingredients may present lower or more variable protein bioaccessibility and AA digestibility than FM in *P. vannamei* [64,65]. Such variability may reflect raw material composition and processing conditions, both of which can alter protein structure and AA availability.

Despite the significant differences observed in vitro, final body weight, SGR, and survival remained similar among dietary treatments. Higher protein bioaccessibility, therefore, did not necessarily translate into improved in vivo performance under the evaluated BFT conditions. One possible explanation is that nutrients and microbial biomass from the biofloc partially buffered differences in dietary nutrient availability, as suggested by previous BFT studies [66,67]. Our previous work similarly showed that the nutritional importance of biofloc as a natural protein source can increase when dietary protein supply is reduced [14], while extracellular enzymes and other bioactive compounds associated with the biofloc may contribute to nutrient digestion and utilization in vivo [68]. However, this mechanism cannot be demonstrated in the present study because biofloc consumption and nutrient assimilation were not directly quantified. Moreover, its nutritional contribution depends on carbon source, the C:N ratio, biofloc composition, and shrimp grazing activity [14,69]. Therefore, in vitro digestibility remains useful for screening diets but should be interpreted together with in vivo performance, particularly under BFT.

The lower apparent FCR values observed with A7.5 and AP7.5 diets despite their comparatively low or intermediate in vitro protein bioaccessibility further indicate that total AA release was not the sole determinant of apparent feed utilization. Instead, the overall nutritional balance of the diets, particularly their AA profiles, may have contributed to the observed response. In juvenile *P. vannamei*, differences in protein source and AA profile can affect protein synthesis, degradation, and retention [70]. Similarly, dietary lysine levels influence feed efficiency, PER, and protein deposition, even when diets contain comparable crude protein levels [71]. Therefore, the slightly lower proportions of lysine and branched-chain amino acids in the plant-based diets may have contributed to differences in apparent protein utilization. A7.5 and AP7.5 also showed higher apparent protein and energy retention efficiencies than AP0, although these differences were not significant. Thus, the biological value and AA balance of dietary protein may have been more relevant to apparent feed utilization than total protein bioaccessibility alone.

From a practical perspective, A7.5 and AP7.5 provided the most favorable combined feed utilization and economic response. Both diets showed the lowest apparent FCR and ECR values, reducing feed cost by approximately 1.12 € per kilogram of shrimp biomass relative to AP0 while maintaining growth performance comparable to the Control. Therefore, complete FM removal did not provide additional biological or economic advantages under the evaluated BFT conditions. Instead, retaining 7.5% FM in combination with animal and mixed animal- and plant-derived protein sources provided the most favorable overall balance between apparent feed utilization, feed cost, and reduced dependence on marine ingredients. This agrees with previous work in super-intensive BFT systems, where a diet containing 12.5% FM together with SBM and meat and viscera meals resulted in a higher final shrimp weight than diets containing either 0% or 21% FM, although FCR and survival remained unaffected [72]. Similarly, FM was successfully reduced from 30% to 6% using a co-extruded SBM and poultry by-product meal blend without compromising shrimp growth or feed efficiency [73].

Beyond apparent feed utilization, the absence of significant effects on final body weight, SGR, and survival indicates that reducing or completely removing FM did not compromise shrimp growth during the 74-day trial. This finding is consistent with previous studies showing that complete or near complete FM replacement can be feasible when alternative formulations are appropriately balanced to meet the nutritional requirements of the species [74,75]. For instance, in shrimp of similar initial size, a diet combining poultry by-product meal, Antarctic krill meal, and corn gluten meal supported growth equivalent to an FM-based reference diet [76]. Likewise, the 15% FM Control did not outperform several low or FM-free diets in the present study, supporting the view that high FM inclusion is not necessary to maintain growth when diets are appropriately formulated [77]. Nevertheless, the poorer apparent FCR and ECR of AP0 showed that maintaining growth alone does not imply equivalent feed or economic efficiency, highlighting the importance of evaluating several performance criteria simultaneously.

Shrimp proximate composition remained stable across dietary treatments, further demonstrating the capacity of *P. vannamei* to maintain tissue composition when fed contrasting protein sources. This agrees with previous studies reporting little change in whole-body composition after moderate FM substitution [78,79], although effects may emerge at higher replacement levels depending on the ingredient used [80,81]. In the present study, the higher energy and lower protein content of initial shrimp relative to final animals occurred across treatments and were therefore more likely related to somatic development than dietary formulation [82]. Biofloc composition, in contrast, responded more strongly to diet. Biofloc associated with P0, P7.5, and AP0 contained higher CP and energy, whereas those associated with A0 and A7.5 showed higher ash contents. These differences indicate that feed composition influenced nutrient fluxes and microbial metabolism within the BFT system. The greater protein bioaccessibility of plant-based diets may have increased the availability of nitrogenous substrates to the microbial community, thereby favoring more protein rich biofloc [83]. However, the higher CP content of the biofloc was not associated with superior growth or apparent feed utilization. Differences in AA composition, digestibility, microbial biomass, or ingestion rate could all influence the effective contribution of the floc to shrimp nutrition. Because biofloc AA profiles and microbial functional activity were not characterized, the nutritional significance of these compositional differences remains unresolved.

Microbial diversity analyses identified temporal succession as the main driver of both biofloc and intestinal microbiota structure. Significant changes in both α- and β-diversity occurred between the beginning and the end of the experiment, whereas the effects of dietary treatments were more evident at the level of community composition and individual bacterial families than in overall richness or evenness. Biofloc richness and diversity declined over time, consistent with progression from a heterogeneous early community toward a more specialized consortium adapted to nutrient recycling [84]. In contrast, intestinal microbial diversity increased with shrimp development and continuous exposure to dietary substrates and biofloc-associated microorganisms [20]. β-diversity analyses further indicated that these temporal changes involved both compositional shifts and changes within group dispersion, confirming ecological succession in both compartments. This ecological succession was accompanied by distinct taxonomic trajectories. Early communities showed higher abundance of *Rhodobacteraceae*, *Pseudoalteromonadaceae*, *Fusobacteriaceae*, and *Fusibacteraceae*, taxa associated with nutrient cycling, organic matter degradation, microbial turnover, and probiotic activity [16,18,21,85,86,87]. As the system matured, biofloc became enriched in *Caldilineaceae*, *Rhizobiaceae*, *Woesebacteria*, and *Pirellulaceae*, groups associated with carbon, nitrogen, and phosphorus cycling under heterogeneous oxygen conditions [88,89,90]. The increase in *Thermoanaerobaculaceae* may also indicate localized microoxic niches within the biofloc matrix [89]. The intestinal microbiota followed a distinct trajectory driven by both environmental exposure and host selection [91]. Early communities were dominated by *Rhodobacteraceae* and *Flavobacteriaceae*, families previously associated with early shrimp core microbiome [21,92], together with generalists *Halieaceae*, *Pirellulaceae*, and *Woeseiaceae*. By the end of the experiment, the intestinal community showed higher abundances of *Microbacteriaceae*, *Gemmataceae*, *Ilumatobacteraceae*, and *Segniliparaceae*, likely introduced through sustained biofloc ingestion. *Microbacteriaceae* has been linked to growth promotion and resistance to *Vibrio parahaemolyticus* [93]. The relative stable abundance of *Vibrionaceae* and *Shewanellaceae* across the trial period further indicates that these families were not strongly affected by the dietary treatments [94].

Dietary formulation nevertheless significantly modulated microbial composition, particularly when diets were grouped according to ingredient source, whereas FM inclusion level alone had comparatively little influence on either the biofloc or intestinal microbiota, consistent with previous observations [95]. Although most dominant taxa were shared across diets, ingredient-based comparisons revealed significant differences in the relative abundance of specific bacterial families. In biofloc, animal-based diets presented higher relative abundance of *Pacebacteria*, *Rhizobiaceae* and *Saccharimonadaceae* and lower *Kiloniellaceae* and WCHB1-41 than plant-based diets. These taxa have been associated with nitrogen transformation, lipid metabolism or denitrification [96], although functional consequences cannot be inferred because microbial activity was not directly measured. In the intestine, animal-based diets showed higher abundance of *Flavobacteriaceae* and *Shewanellaceae*, taxa commonly associated with gut health and resistance to *Vibrio* infections [97,98], whereas plant-based diets showed higher *Halieaceae* and *Microbacteriaceae*, previously reported in biofloc during mid-culture stages and associated with beneficial effects on shrimp health as discussed above [89]. These results confirm that dietary protein sources significantly affected selected bacterial families, but not overall diversity, and should be interpreted as evidence of broad changes in intestinal health.

Direct comparison between biofloc and shrimp intestinal microbiota revealed only a limited family-level overlap despite continuous environmental exposure, indicating that the intestinal community was not a passive reflection of the surrounding biofloc but was strongly shaped by host-mediated selection and specific habitat conditions [99]. The limited shared core is compatible with some microbial exchange, while temporal succession and ecological niche remain stronger determinants of community assembly than dietary formulation.

Taken together, these findings highlight the importance of evaluating alternative protein formulations within the ecological framework of BFT rather than considering the formulated feed in isolation. The favorable response of A7.5 and AP7.5 reflects their combined productive and economic performance, while the microbiome results show that protein source can modulate selected bacterial groups without overriding the stronger effects of temporal development and habitat. Future research should directly quantify biofloc consumption and its nutritional contribution, while integrating amino acid profiling, stable isotope analysis, and microbial functional characterization. These approaches would clarify the mechanisms linking diet formulation, biofloc nutritional value, microbial dynamics and shrimp performance, thereby supporting the development of more precise and sustainable feeding strategies for BFT-based shrimp production.

## 5. Conclusions

The present study demonstrates that complete fishmeal replacement was not fully supported by the alternative ingredients evaluated. In contrast, reducing fishmeal inclusion by 50% relative to the Control diet (15% FM) and complementing it with either animal protein sources or a combination of animal- and plant-derived proteins provided the most favorable overall balance between growth performance, apparent feed utilization, protein efficiency, feed cost, and reduced reliance on marine ingredients. Plant-based diets exhibited higher in vitro protein digestibility and AA release, but this advantage was not translated into improved growth, indicating that in vitro protein bioaccessibility alone was insufficient to predict in vivo productive performance under BFT. Biofloc-derived nutrients may have contributed to buffering dietary differences, although their contribution was not directly quantified.

Microbial community structure was primarily driven by temporal succession associated with biofloc maturation and shrimp development, while dietary protein source modulated the abundance of specific bacterial families. The limited taxonomic overlap between biofloc and intestinal communities further suggests strong habitat- and host-mediated selection. Collectively, these findings demonstrate that diets containing 7.5% fishmeal supplemented with either animal or mixed animal- and plant-derived protein sources are sufficient to sustain optimal performance of juvenile *Penaeus vannamei* under BFT conditions, maintaining productive performance while improving apparent feed and economic efficiency.

## Figures and Tables

**Table 3:** Water quality mean values during the experimental phase (mean ± SE).

Water Quality Parameter	Juvenile Phase
pH	8.02 ± 0.01
Oxygen (mg/L)	6.38 ± 0.01
Salinity (g/L)	20.25 ± 0.01
Temperature (°C)	28.67 ± 0.02
Ammonia (mg/L)	0.22 ± 0.03
Nitrite (mg/L)	0.31 ± 0.04
Nitrate (mg/L)	52.37 ± 7.67
Phosphorus (mg/L)	4.22 ± 0.78
SSTs (mg/L)	360.40 ± 9.96
Alkalinity (mg/L)	168.08 ± 1.74

**Table 4:** Final values of protein hydrolysis (mg AA released/100 mg of feed) and bioavailable protein content (%) of the tested feeds (A7.5, A0, P7.5, P0, AP7.5, AP0, and Control) (mean ± SE, *n* = 3). The values within the same column with different superscripts are significantly different at (*p* < 0.05). Newman–Keuls post hoc test.

Dietary Formulations	mg AA Released/ 100 mg of Feed	Bioavailable Protein (%)
A7.5	17.44 ± 0.37 **^d^**	49.81 ± 1.06 **^d^**
A0	16.63 ± 0.26 **^d^**	47.95 ± 0.75 **^d^**
P7.5	23.70 ± 0.19 **^b^**	66.11 ± 0.52 **^b^**
P0	27.60 ± 2.42 **^a^**	76.69 ± 6.73 **^a^**
AP7.5	19.05 ± 0.33 **^cd^**	53.91 ± 0.93 **^cd^**
AP0	18.94 ± 0.50 **^cd^**	53.46 ± 1.42 **^cd^**
Control	22.34 ± 0.78 **^bc^**	62.58 ± 2.20 **^bc^**

**Table 5:** Growth performance of *P. vannamei* cultured using different tested feeds (A7.5, A0, P7.5, P0, AP7.5, AP0 and Control), (mean ± SE, *n* = 3). The values within the same column with different superscripts are significantly different at (*p* < 0.05). Newman–Keuls post hoc test. SGR: specific growth rate, FI: feed intake, FCR: feed conversion ratio, PIR: protein intake ratio, PER: protein efficiency ratio.

Treatment	Initial Weight (g)	Final Weight (g)	SGR (%/Day)	FI (g/100g ·Day)	FCR	PIR	PER	Survival (%)	Protein Retention (%)	Energy Retention (%)
A7.5	2.58 ± 0.12	8.29 ± 0.55	1.57 ± 0.09	2.80 ± 0.12	2.08 ± 0.17 **^b^**	0.98 ± 0.04	1.39 ± 0.10 **^a^**	83.00 ± 3.51	23.17 ± 4.16	11.05 ± 2.14
A0	2.53 ± 0.21	7.41 ± 0.16	1.46 ± 0.11	3.02 ± 0.10	2.39 ± 0.11 **^ab^**	1.05 ± 0.03	1.21 ± 0.05 **^ab^**	82.67 ± 1.20	21.54 ± 2.16	10.50 ± 1.19
P7.5	2.57 ± 0.10	7.57 ± 0.41	1.46 ± 0.04	3.01 ± 0.13	2.38 ± 0.15 **^ab^**	1.08 ± 0.04	1.18 ± 0.07 **^ab^**	82.33 ± 0.67	23.31 ± 2.46	11.46 ± 1.37
P0	2.51 ± 0.18	7.22 ± 0.34	1.43 ± 0.09	3.12 ± 0.15	2.51 ± 0.16 **^ab^**	1.12 ± 0.05	1.12 ± 0.07 **^ab^**	82.67 ± 0.33	19.22 ± 2.56	8.63 ± 1.30
AP7.5	2.58 ± 0.08	8.20 ± 0.32	1.56 ± 0.07	2.82 ± 0.07	2.11 ± 0.12 **^b^**	1.00 ± 0.03	1.35 ± 0.08 **^ab^**	81.67 ± 0.33	22.20 ± 2.02	10.06 ± 1.13
AP0	2.55 ± 0.13	6.74 ± 0.40	1.31 ± 0.09	3.25 ± 0.15	2.85 ± 0.12 **^a^**	1.15 ± 0.05	1.01 ± 0.09 **^b^**	81.00 ± 0.00	14.91 ± 0.03	6.45 ± 0.05
Control	2.57 ± 0.07	7.55 ± 0.33	1.45 ± 0.02	2.97 ± 0.11	2.35 ± 0.11 **^ab^**	1.06 ± 0.04	1.19 ± 0.06 **^ab^**	82.33 ± 0.88	23.18 ± 2.40	10.71 ± 1.24

**Table 6:** Shrimp and biofloc composition of *P. vannamei* cultured under BFT using different ingredients in diet (A7.5, A0, P7.5, P0, AP7.5, AP0 and Control) (mean ± SE, *n* = 3). The values within the same column with different superscripts are significantly different at *p* < 0.05. The lowercase letters refer to differences between treatments at the end of the experiment. The (*) symbol refers to temporal differences between the beginning and the end of the experiment at *p* < 0.05. Newman–Keuls post hoc test. DM (%): dry matter, Ashes (%), CP (%): crude protein, OM (%): organic matter, GE (MJ/kg): gross energy.

Sample	Treatment	DM	Ashes	CP	GE
**Shrimp**	**Initial**	23.13 ± 0.47	14.12 ± 0.48	72.82 ± 0.04	20.84 ± 0.02
	A7.5	17.79 ± 1.87	16.91 ± 1.81	75.61 ± 0.92 *	19.66 ± 0.24 *
	A0	19.18 ± 0.32	17.63 ± 0.40	73.55 ± 0.59	19.44 ± 0.29 *
	P7.5	19.90 ± 0.40	17.22 ± 0.07	75.10 ± 0.32 *	19.84 ± 0.15 *
	P0	18.52 ± 1.58	17.37 ± 1.61	75.56 ± 0.36 *	19.78 ± 0.10 *
	AP7.5	17.79 ± 1.31	17.62 ± 0.62	75.36 ± 0.14 *	19.35 ± 0.13 *
	AP0	17.24 ± 1.50	17.70 ± 0.39	74.79 ± 0.10 *	19.43 ± 0.09 *
	Control	19.55 ± 0.73	17.10 ± 0.84	75.73 ± 0.23 *	19.83 ± 0.11 *
		**OM**	**Ashes**	**CP**	**GE**
**Biofloc**	**Initial**	45.48 ± 0.31	47.00 ± 0.37	26.05 ± 0.28	12.50 ± 0.07
	A7.5	46.77 ± 0.62 **^c^**	43.30 ± 1.43 **^a^**	28.24 ± 0.34 **^b^**	12.23 ± 0.35 **^c^**
	A0	47.29 ± 0.87 **^c^**	43.74 ± 1.54 **^a^**	28.67 ± 1.18 **^b^**	12.41 ± 0.34 **^bc^**
	P7.5	52.13 ± 0.93 ^ab^*	37.22 ± 1.69 ^b^*	33.65 ± 0.90 ^a^*	13.61 ± 0.38 **^a^**
	P0	52.70 ± 0.68 ^a^*	36.26 ± 1.11 ^b^*	33.83 ± 0.97 ^a^*	13.82 ± 0.30 **^a^**
	AP7.5	49.92 ± 0.31 ^b^*	40.74 ± 0.82 ^ab^*	30.34 ± 0.64 ^b^*	13.08 ± 0.03 **^abc^**
	AP0	51.65 ± 0.28 ^ab^*	37.63 ± 0.71 ^b^*	32.92 ± 0.39 ^a^*	13.59 ± 0.15 **^a^**
	Control	51.35 ± 0.29 ^ab^*	38.04 ± 0.55 ^b^*	33.18 ± 0.18 ^a^*	13.36 ± 0.10 **^ab^**

**Table 7:** α diversity characterization of biofloc and shrimp intestine samples at family level (mean ± SE, *n* = 3). To aid interpretation, diets were summarized according to temporal stage (Initial and Final), ingredient base (Animal, Plant, Mixed), fishmeal inclusion level (0 and 7.5%), and individual dietary treatments (A7.5, A0, P7.5, P0, AP7.5, AP0, and Control). The values within the same column with different superscripts are significantly different at (*p* < 0.05). Bonferroni post hoc test.

	Chao1	Simpson	Shannon
**Biofloc**			
Initial	125.00 ± 7.22 **^a^**	0.94 ± 0.05	3.43 ± 0.20 **^a^**
Final	83.90 ± 2.02 **^b^**	0.89 ± 0.01	2.92 ± 0.05 **^b^**
Animal	80.33 ± 2.32	0.91 ± 0.01	2.98 ± 0.06
Plant	89.17 ± 4.60	0.89 ± 0.02	2.94 ± 0.08
Animal–Plant Mix	80.33 ± 4.26	0.87 ± 0.02	2.78 ± 0.12
7.5% FM	84.33 ± 3.70	0.88 ± 0.02	2.86 ± 0.09
0% FM	82.22 ± 2.99	0.90 ± 0.01	2.94 ± 0.05
A7.5	83.67 ± 3.38	0.91 ± 8.82 × 10^−3^	2.97 ± 0.09
A0	77.00 ± 2.08	0.91 ± 0.01	2.99 ± 0.16
P7.5	92.67 ± 8.29	0.89 ± 0.02	2.94 ± 0.13
P0	85.67 ± 4.98	0.89 ± 0.03	2.95 ± 0.12
AP7.5	76.67 ± 4.48	0.84 ± 0.05	2.68 ± 0.22
AP0	84.00 ± 7.57	0.90 ± 0.02	2.88 ± 0.10
Control	87.67 ± 1.45	0.91 ± 5.77 × 10^−3^	3.04 ± 0.01
**Shrimp intestine**			
Initial	88.17 ± 2.99	0.88 ± 7.92 × 10^−3^ **^b^**	2.92 ± 0.03 **^b^**
Final	86.24 ± 1.84	0.92 ± 2.95 × 10^−3^ **^a^**	3.20 ± 0.02 **^a^**
Animal	86.25 ± 3.90	0.92 ± 0.01	3.18 ± 0.07
Plant	83.17 ± 3.46	0.93 ± 3.28 × 10^−3^	3.22 ± 0.03
Animal–Plant Mix	90.00 ± 3.02	0.92 ± 4.60 × 10^−3^	3.18 ± 0.04
7.5% FM	86.11 ± 14.97	0.92 ± 0.02	3.19 ± 0.21
0% FM	86.83 ± 8.61	0.92 ± 0.02	3.20 ± 0.13
A7.5	88.17 ± 6.26	0.91 ± 0.01	3.14 ± 0.12
A0	84.33 ± 5.12	0.92 ± 0.01	3.23 ± 0.08
P7.5	80.50 ± 6.23	0.93 ± 6.32 × 10^−3^	3.20 ± 0.06
P0	85.83 ± 3.25	0.93 ± 2.24 × 10^−3^	3.23 ± 0.02
AP7.5	89.67 ± 6.22	0.93 ± 6.01 × 10^−3^	3.24 ± 0.06
AP0	90.33 ± 1.14	0.91 ± 5.43 × 10^−3^	3.21 ± 0.03
Control	84.83 ± 4.96	0.93 ± 1.67 × 10^−3^	3.23 ± 0.02

**Table 8:** Summary of permutational multivariate analysis of variance (PERMANOVA), homogeneity of multivariate dispersion analysis (PERMDISP), and pairwise comparisons for microbial community structure in biofloc and shrimp intestinal samples. PERMANOVA was performed using Euclidean distances calculated from CLR-transformed relative abundance data. Pairwise comparisons are reported only when they remain significant after correction for multiple testing (*n* = 3).

Sample	Factor	PERMANOVA			PERMDISP	Significant Pairwise Comparisons
		F-Value	R^2^	*p*-Value		
**Biofloc**	Time	13.834	0.386	0.002	0.001	Initial vs. Final
	Dietary Treatment	1.4793	0.388	0.001	0.883	None after correction
	Ingredient source	1.8752	0.248	0.001	0.827	Animal vs. Plant; Animal vs. Mixed; Control vs. Mixed
	FM inclusion	1.4316	0.137	0.021	0.115	None after correction
**Shrimp**	Time	11.813	0.204	0.001	0.001	Initial vs. Final
	Dietary Treatment	1.3868	0.192	0.007	0.439	AP0 vs. A0; AP0 vs. A7.5; AP0 vs. P0; AP0 vs. P7.5
	Ingredient source	1.6147	0.113	0.005	0.922	Plant vs. Animal; Plant vs. Mixed
	FM inclusion	1.0524	0.051	0.328	0.180	Not applicable

## Data Availability

The original data presented in the study are openly available in institutional repository RiuNet (Universitat Politècnica de València) at https://riunet.upv.es/handle/10251/238976 (accessed on 1 September 2026).

## Supplementary Materials

The following supporting information can be downloaded at: https://www.mdpi.com/article/10.3390/cimb48090904/s1.

## Abbreviations

The following abbreviations are used in this manuscript:

AA	Amino acid
ANFs	Anti-nutritional factors
ALA	Alanine
ARG	Arginine
ASP	Aspartic acid
BFT	Biofloc technology
C	Carbon
CBM	Soybean meal
CL	Crude lipid
CLR	Centered-log ratio
CP	Crude protein
CYS	Cysteine
DM	Dry matter
DO	Dissolved oxygen
EAA	Essential AA
ECR	Economic conversion ratio
FCR	Feed conversion ratio
FI	Feed intake
FM	Fishmeal
GE	Gross energy
GLU	Glutamic acid
GLY	Glycine
HIS	Histidine
ILE	Isoleucine
LEU	Leucine
LYS	Lysine
MET	Methionine
N	Nitrogen
NEAA	Non-essential AA
OM	Organic matter
PHE	Phenylalanine
PIR	Protein intake ratio
PER	Protein efficiency ratio
PL	Post-larvae
PRO	Proline
SE	Standard error
SER	Serine
SGR	Specific growth rate
THR	Threonine
TYR	Tyrosine
UAL	Universidad de Almería
UPV	Universitat politècnica de València
VAL	Valine
