# Supplementary material for: Combined Animal and Plant Protein Enable High Fishmeal Replacement in Penaeus vannamei Under Biofloc Technology
Source: Curr Issues Mol Biol. 2026 Sep 4;48(9):904. doi: 10.3390/cimb48090904 (PMC13604989; doi:10.3390/cimb48090904)
Supplement: Supplementary file 1 [file cimb-48-00904-s001.zip › cimb-4432944-supplementary.pdf]

SUPPLEMENTARY MATERIAL

Table S1: Water quality parameters in both experimental phases for *P.vannamei* cultivation with different diets (mean ± S.E; n=3). The values within the same column with different superscripts are significantly different at ($p < 0.05$). *Kruskal-wallis* test and *Bonferroni* posthoc test.

	**A7.5**	**A0**	**P7.5**	**P0**	**AP7.5**	**AP0**	**Control**
pH	8.02±0.02	8.04±0.02	8.01±0.02	8.02±0.02	8.02±0.02	8.01±0.02	8.00±0.02
Oxygen (mg/L)	6.39±0.02 $^{\mathbf{ab}}$	6.45±0.02 **a**	6.36±0.02 **b**	6.38±0.02 $^{\mathbf{ab}}$	6.37±0.02 $^{\mathbf{ab}}$	6.34±0.02 **b**	6.42±0.01 $^{\mathbf{ab}}$
Salinity (g/L)	20.33±0.05 $^{\mathbf{ab}}$	20.34±0.04 **a**	20.23±0.03 $^{\mathbf{ab}}$	20.22±0.03 $^{\mathbf{ab}}$	20.18±0.05 **b**	20.31±0.03 **a**	20.17±0.03 $^{\mathbf{ab}}$
Temperature (ºC)	28.58±0.06 $^{\mathbf{ab}}$	28.70±0.07 $^{\mathbf{ab}}$	28.89±0.07 $^{\mathbf{a}}$	28.69±0.06 $^{\mathbf{ab}}$	28.56±0.06 $^{\mathbf{b}}$	28.60±0.06 $^{\mathbf{ab}}$	28.64±0.06 $^{\mathbf{ab}}$
Ammonia (mg/L)	0.21±0.07	0.20±0.06	0.22±0.07	0.22±0.07	0.21±0.07	0.25±0.07	0.21±0.07
Nitrite (mg/L)	0.30±0.11	0.32±0.11	0.28±0.10	0.28±0.10	0.35±0.14	0.34±0.13	0.29±0.11
Nitrate (mg/L)	49.07±21.56	49.11±20.54	52.94±21.79	52.86±21.98	52.76±22.38	53.03±21.94	56.86±23.35
Phosphorus (mg/L)	4.07±1.95	6.10±3.48	2.84±1.23	3.21±1.40	5.61±2.70	4.29±2.06	3.95±1.50
TSSs (mg/L)	339.12±23.72	401.39±34.53	359.44±26.96	346.49±23.97	373.64±26.71	323.56±21.46	378.27±24.94
Alkalinity (mg/L)	172.94±3.85 $^{\mathbf{ab}}$	170.98±3.80 $^{\mathbf{ab}}$	161.40±4.30 $^{\mathbf{ab}}$	155.12±3.65 $^{\mathbf{b}}$	174.98±5.50 $^{\mathbf{a}}$	179.62±5.44 $^{\mathbf{a}}$	161.50±4.55 $^{\mathbf{ab}}$

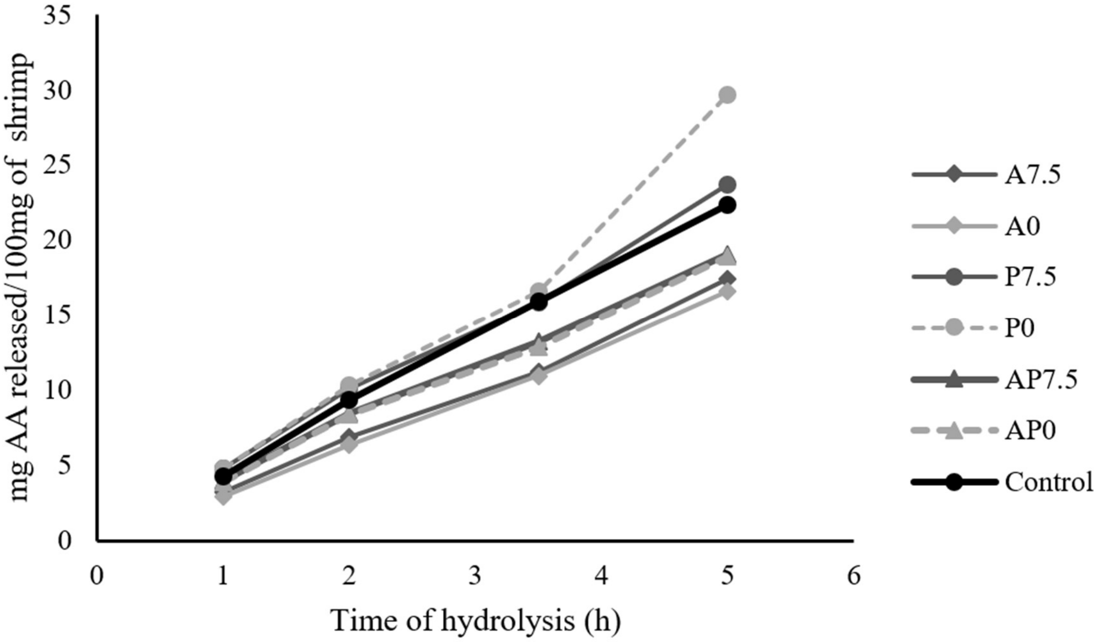

Figure S1: *In vitro* simulation of the shrimp digestion of the protein fraction present in the tested feeds (A7.5, A0, P7.5, P0, AP7.5, AP0, and Control) with mg of amino acids per 100mg of shrimp, released during time (h). Error bars correspond to the standard error.

Table S2: Summary of the quality of 16S rRNA gene sequencing of biofloc and shrimp intestine samples at the beginning and end of the experiment.

MAIN EFFECTS	Input	Filtered	Input passed filter (%)	Denoised	Non-chimeric	Input non-chimeric (%)
**Biofloc**						
Initial	97853.00±5649.37	81542.00±4707.71	83.33±0.01	79298.00±4578.39	79298.00±4578.39	81.04±0.01
A7.5	64226.00±7690.56	61364.00±7324.67	95.55±0.07	60047.70±7328.59	60043.00±7329.58	93.44±0.22
A0	79544.7±2913.90	67350.00±2730.34	95.48±0.07	65942.30±2686.65	65926.70±2670.98	93.46±0.13
P7.5	67990.70±9762.72	64450.00±9912.82	94.45±1.17	63070.00±9823.66	63070.00±9823.66	92.37±1.37
P0	59193.70±9792.63	56420.70±9598.64	95.16±0.81	55107.30±9631.39	55107.30±9631.39	92.81±1.04
AP7.5	62212.00±3887.74	56777.00±3845.61	91.41±4.00	55581.30±3766.90	55581.30±3766.90	89.49±4.00
AP0	70623.00±6322.36	67468.30±6234.44	95.51±0.20	66181.00±6143.05	66141.70±6108.94	93.63±0.15
Control	55963.7±5596.61	52241.30±4721.79	93.54±0.98	50855.70±4670.29	50855.70±4670.29	91.03±0.82
Total	68575.8±3238.05	63451.70±2640.45	93.05±0.93	62010.40±2595.87	62003.00±2594.40	90.91±0.95
**Shrimp intestine**						
Initial	76936.50±9976.52	72842.70±9803.79	94.34±1.08	71318.50±9775.11	71318.50±9775.11	92.20±1.23
A7.5	66559.70±13308.70	62711.30±12722.20	94.05±1.01	61165.30±12632.30	61165.30±12632.30	91.22±1.11
A0	51282.20±13181.2	48719.30±12589.40	94.90±0.45	47252.80±12490.20	47252.80±12490.20	91.13±0.94
P7.5	48903.70±10299.90	45603.20±9538.14	93.76±1.08	44162.80±9419.17	44158.80±9418.25	89.94±0.96
P0	66755.50±8174.64	62808.00±7322.42	94.45±1.07	61328.70±7255.62	61322.80±7260.34	92.07±1.00
AP7.5	77132.50±15029.40	73276.30±14405.80	94.78±0.56	71651.70±14299.80	71644.00±14299.40	91.75±1.31
AP0	80319.50±9781.09	74884.20±9093.24	93.32±0.73	73195.30±9106.58	73190.70±9107.63	91.04±0.74
Control	62062.50±15756.50	58979.50±15131.3	94.69±0.63	57425.20±15059.60	57425.20±15059.60	91.55±0.98
Total	66244.00±4295.92	62478.10±4082.11	94.29±0.29	60937.50±4056.62	60934.80±4056.67	91.36±0.36

Table S3: Family-level microbial composition of biofloc associated with shrimp fed experimental diets, grouped by sampling time point (Initial and Final). The values within the same row with different superscripts are significantly different at ($p < 0.05$).

Biofloc-Family level	Initial	Final
Caldilineaceae	4.00±0.00 [b]	808.90±393.39 [a]
Fusibacteraceae	9505.00±549.06[a]	0 [b]
Fusobacteriaceae	738.00±426.08[a]	0 [b]
Kiloniellaceae	701.00±40.41	597.95±73.92 [a]
Legionellaceae	76.00±4.62 [b]	2404.81±403.89 [a]
Pacebacteria	0 [b]	1252.57±487.36 [a]
Pirellulaceae	3535.00±203.80 [b]	7965.05±640.48 [a]
Pseudoalteromonadaceae	9629.00±555.99 [a]	43.33±28.24 [b]
Psychromonadaceae	4215.00±243.06 [a]	325.71±68.77 [b]
Rhizobiaceae	1207.00±69.86 [b]	776.24±111.30 [a]
Rhodobacteraceae	7197.00±415.69 [a]	1739.90±333.86 [b]
Saccharimonadaceae	89.00±5.20 [b]	2143.52±642.54 [a]
SAR324_clade (Marine_group_B)	1311.00±75.63 [b]	4840.67±847.70 [a]
Stappiaceae	9.00±0.58 [b]	1030.71±404.33 [a]
Thermoanaerobaculaceae	131.00±7.51 [b]	3092.19±534.45 [a]
WCHB1-41	515.00±29.44 [b]	880.00±210.28 [a]
Woesebacteria	24.00±1.15 [b]	9299.90±1626.99 [a]

Table S4: Family-level microbial composition of shrimp fed experimental diets, grouped by sampling time point (Initial and Final). The values within the same row with different superscripts are significantly different at ($p < 0.05$).

Shrimp – Family level	Initial	Final
A4b	221.83±31.05 [b]	7018.93±692.12 [a]
AKYG1722	568.33±83.35	763.19±60.58
Flavobacteriaceae	4945.50±779.39 [a]	965.10±109.24 [b]
Gemmataceae	193.17±31.80 [b]	1554.48±160.24 [a]
Halieaceae	3603.83±557.32 [a]	764.29±76.84 [b]
Ilumatobacteraceae	481.00±91.75 [b]	1302.00±129.19 [a]
Microbacteriaceae	135.67±15.81 [b]	1264.67±146.58 [a]
Mycobacteriaceae	635.33±80.77	814.90±72.26
Nocardiaceae	197.50±32.03	743.29±245.27
Pirellulaceae	20511.00±2718.43 [a]	9864.02±782.36 [b]
Psychromonadaceae	12.33±10.89	873.52±418.17
Rhodobacteraceae	7939.00±1147.71 [a]	3872.48±338.91 [b]
Segniliparaceae	0.67±0.67 [b]	807.98±159.24 [a]
Shewanellaceae	661.67±632.32	354.05±122.46
Thermoanaerobaculaceae	621.83±87.81 [b]	4712.64±477.63 [a]
Vibrionaceae	378.00±157.62	710.29±149.98
Woeseiaceae	5465.50±767.95 [a]	3008.31±286.06 [b]

Table S5: Taxonomic groups (family level) with more than (1% abundance) mist shared between treatments in biofloc and shrimp intestine samples at the end of the juvenile phase.

	Common presence	Family group
Biofloc	Animal-Plant-Mix-Control	*Woesebacteria, Pirellulaceae, SAR324 clade (Marine_group_B), Thermoanaerobaculaceae, Legionellaceae, Rhodobacteraceae, Rhizobiaceae, Kiloniellaceae*
	Plant-Mix-Control	*WCHB1-41, Caldilineaceae*
	Animal-Plant-Mix	*Saccharimonadaceae*
	Animal-Mix	*Pacebacteria, Stappiaceae*
Shrimp	Animal-Plant-Mix-Control	*Pirellulaceae, A4b, Thermoanaerobaculaceae, Rhodobacteraceae, Woeseiaceae, Gemmataceae, Ilumatobacteraceae, Psychromonadaceae, Mycobacteriaceae, Segniliparaceae, AKYG1722, Halieaceae, Flavobacteriaceae*
	Plant-Mix-Control	*Microbacteriaceae*
	Animal-Plant-Mix	*Vibrionaceae, JG30-KF-CM45*
	Plant-Control	*Nocardiaceae*
	Animal	*Shewanellaceae*

Table S6: Family-level microbial composition of biofloc associated with shrimp fed experimental diets, grouped by dietary ingredient source (Animal, Plant, Mixed, and Control). The values within the same row with different superscripts are significantly different at ($p < 0.05$).

**Biofloc – Family level**	**Animal**	**Plant**	**MIX**	**Control**
Caldilineaceae	77.83±24.76	2038.50±1270.18	558.50±300.35	312.67±156.74
Kiloniellaceae	305.33±40.96 **b**	806.67±131.77 **a**	554.00±154.42 **ab**	853.67±64.75 **ab**
Legionellaceae	3246.50±1118.46	1912.83±543.43	2690.50±498.81	1134.00±777.11
Pacebacteria	3321.67±1431.94 **a**	166.67±49.23 **b**	652.17±213.09 **ab**	487.00±145.50 **ab**
Pirellulaceae	9138.00±672.86	7918.33±1599.78	7881.00±1247.41	5880.67±1522.25
Rhizobiaceae	1020.17±183.35 **a**	952.50±288.64 **ab**	425.17±78.14 **b**	638.00±169.05 **ab**
Rhodobacteraceae	1498.50±395.58	1564.00±285.93	2247.17±1082.37	1560.00±625.29
Saccharimonadaceae	3332.83±850.49 **a**	771.33±311.21 **ab**	3300.17±1911.20 **ab**	196.00±62.16 **b**
SAR324_clade (Marine_group_B)	5583.67±1633.88	2647.17±1625.93	6487.17±1722.68	4448.67±863.72
Stappiaceae	1066.17±233.44	289.00±106.40	2027.67±1368.54	449.33±189.77
Thermoanaerobaculaceae	2441.83±709.44	3631.67±1195.75	2668.17±500.13	4162.00±2759.33
WCHB1-41	98.00±26.83 **b**	1058.83±262.40 **a**	853.83±314.57 **ab**	2138.67±903.49 **ab**
Woesebacteria	6282.67±2262.53	10260.83±3747.62	12113.83±3486.82	7784.67±3097.68

Table S7: Family-level intestinal microbial composition of shrimp fed experimental diets, grouped by dietary ingredient source (Animal, Plant, Mixed, and Control). The values within the same row with different superscripts are significantly different at ($p < 0.05$).

Shrimp – Family level	Animal	Plant	MIX	Control
A4b	6270.67±1447.80	6084.92±934.83	8809.92±1180.02	6801.50±2536.87
AKYG1722	739.83±128.82	604.25±88.67	971.92±102.77	710.33±168.12
Flavobacteriaceae	1173.92±176.33 **a**	624.25±150.95 **b**	1346.83±248.50 **ab**	465.67±64.29 **ab**
Gemmataceae	1608.75±325.76	1176.17±192.16	2064.67±355.99	1182.17±310.70
Halieaceae	464.00±88.48 **b**	796.83±98.22 **a**	1006.58±180.30 **ab**	815.17±241.84 **ab**
Ilumatobacteraceae	1603.33±354.12	840.75±109.79	1593.00±161.26	1039.83±264.18
JG30-KF-CM45	635.33±111.05	511.33±68.46	910.92±115.65	536.17±137.77
Microbacteriaceae	357.92±104.39 **b**	1662.25±234.80 **a**	1652.17±204.64 **a**	1508.00±542.71 **ab**
Mycobacteriaceae	810.67±106.63	698.08±90.82	907.33±116.00	872.17±380.30
Nocardiaceae	220.83±97.62	952.92±292.90	284.92±119.91	2285.67±1511.19
Pirellulaceae	9372.92±1566.22	8361.33±1052.76	12542.42±1681.52	8494.83±1666.92
Psychromonadaceae	663.17±497.00	499.25±213.99	1695.92±1375.39	398.00±190.96
Rhodobacteraceae	3973.17±744.69	3208.25±468.86	4684.58±705.02	3375.33±710.66
Segniliparaceae	580.08±209.24	1256.83±395.87	523.08±205.60	935.83±500.20
Shewanellaceae	810.42±391.37 **a**	157.42±71.72 **b**	121.58±66.83 **ab**	299.50±153.48 **ab**
Thermoanaerobaculaceae	5108.50±1141.50	3740.25±533.90	5468.33±979.58	4354.33±1090.91
Vibrionaceae	1070.83±426.08	511.33±184.63	682.25±222.90	443.17±203.52
Woeseiaceae	2231.33±435.77	3261.75±495.23	3699.92±657.48	2672.17±631.94

Table S8: Family-level microbial composition of biofloc associated with shrimp fed experimental diets (A7.5, A0, P7.5, P0, AP7.5, AP0, and Control). The values within the same row with different superscripts are significantly different at ($p < 0.05$).

Biofloc-Family level	A7.5	A0	P7.5	P0	AP7.5	AP0	Control
Caldilineaceae	72.00±36.35	83.67±41.34	1394.33±1334.93	2682.67±2422.78	842.33±601.63	274.67±92.34	312.57±156.74
Fusibacteraceae	0	0	0	0	0	0	0
Fusobacteriaceae	0	0	0	0	0	0	0
Kiloniellaceae	284.00±57.19 **b**	326.67±68.27 **ab**	663.00±120.88 **ab**	950.33±227.09 **a**	716.33±273.84 **ab**	391.67±133.74 **ab**	853.67±64.75 **ab**
Legionellaceae	4328.67±2024.06	2164.33±993.39	1606.33±697.48	2219.33±946.65	2332.33±731.65	3048.67±761.87	1134.00±777.11
Pacebacteria	2301.67±1253.77	4341.67±2764.04	218.00±66.00	115.33±71.62	492.33±198.40	812.00±402.66	487.00±145.50
Pirellulaceae	9463.67±598.67	8812.33±1341.36	7491.33±2091.36	8345.33±2870.61	7891.00±1906.43	7871.00±2036.06	5880.67±1522.25
Pseudoalteromonadaceae	0	0	28.00±17.79	10.33±10.33	0	0	265.00±162.56
Psychromonadaceae	219.00±113.01	211.00±135.94	430.67±201.18	274.67±189.45	397.33±241.80	98.67±29.36	648.67±254.09
Rhizobiaceae	1054.33±107.49 **a**	986.00±394.17 **ab**	852.67±406.28 **ab**	1052.33±491.46 **ab**	518.67±141.70 **b**	331.67±41.44 **b**	638.00±169.05 **ab**
Rhodobacteraceae	1446.00±608.55	1551.00±639.78	1236.00±367.20	1892.00±407.86	950.67±220.45	3543.67±2031.77	1560.00±625.29
Saccharimonadaceae	2915.67±1194.46	3750.00±1419.82	1101.00±497.54	441.67±357.82	984.00±455.08	5616.33±3562.55	196.00±62.19
SAR324_clade (Marine_group_B)	3715.00±1353.94	7452.33±2832.44	4219.33±3219.21	1075.00±619.09	3348.00±1551.26	9626.33±1605.38	4448.67±863.72
Stappiaceae	1250.00±445.17	882.33±201.26	365.00±211.40	213.00±78.31	3161.67±2799.21	893.67±492.87	449.33±189.77
Thermoanaerobaculaceae	3146.00±1413.07	1737.67±149.90	3064.33±364.94	4199.00±2587.29	2710.00±801.58	2626.33±778.70	4162.00±2759.33
WCHB1-41	83.67±29.55 **b**	112.33±50.21 **ab**	1188.67±561.00 **ab**	929.00±112.72 **a**	1281.67±526.04 **ab**	426.00±187.13 **ab**	2138.67±903.49 **ab**
Woesebacteria	7078.67±4659.42	5486.67±1803.16	12460.00±6914.61	8061.67±4192.29	14934.33±6345.13	9293.33±3545.93	7784.67±3097.68

Table S9: Family-level intestinal microbial composition of shrimp fed experimental diets (A7.5, A0, P7.5, P0, AP7.5, AP0, and Control). The values within the same row with different superscripts are significantly different at ($p < 0.05$).

Shrimp – Family level	A7.5	A0	P7.5	P0	AP7.5	AP0	Control
A4b	7180.67±2247.16	5360.67±1960.10	5239.17±1715.71	6930.67±784.47	7208.00±1861.91	10411.83±1278.13	6801.50±2536.87
AKYG1722	793.33±217.14	686.33±157.23	532.17±121.55	676.33±133.20	912.50±206.81	1031.33±47.83	710.33±168.12
Flavobacteriaceae	1196.67±202.11	1151.17±309.43	559.17±205.36	689.33±237.46	1301.67±431.66	1392.00±290.79	465.67±64.29
Gemmataceae	1986.17±591.23	1231.33±245.72	993.83±247.78	1358.50±296.28	2440.83±548.59	1688.50±447.27	1182.17±310.70
Halieaceae	514.50±114.27	413.50±142.70	694.83±158.24	898.83±115.10	1231.83±340.06	781.33±84.31	815.17±241.84
Ilumatobacteraceae	1986.50±654.79	1220.17±253.54	794.00±185.69	887.50±132.99	1461.67±260.98	1724.33±198.51	1039.83±264.18
JG30-KF-CM45	722.00±169.99	548.67±149.55	469.33±111.10	553.33±87.04	662.67±102.03	1159.17±154.25	536.17±137.77
Microbacteriaceae	$449.50±168.31^{\mathbf{bc}}$	$266.33±127.54^{\mathbf{c}}$	$1258.33±292.07^{\mathbf{a}}$	$2066.17±303.35^{\mathbf{a}}$	$1308.00±278.52^{\mathbf{ab}}$	$1996.33±243.51^{\mathbf{a}}$	$1508.00±542.71^{\mathbf{abc}}$
Mycobacteriaceae	881.33±154.92	740.00±155.01	676.33±148.32	719.83±118.75	842.00±186.72	972.67±150.46	872.17±380.30
Nocardiaceae	172.50±116.18	269.17±165.82	777.17±467.18	1128.67±383.23	426.50±226.67	143.33±62.21	2285.67±1511.19
Pirellulaceae	11022.00±2840.69	7723.83±1279.08	7560.17±1870.40	9162.50±1058.94	12521.17±2600.80	12563.67±2382.59	8494.83±1666.92
Psychromonadaceae	203.83±71.69	1122.50±998.66	349.33±199.36	649.17±390.84	412.00±171.00	2979.83±2763.13	398.00±190.06
Rhodobacteraceae	4014.17±838.91	3932.17±1317.42	2335.50±551.54	4081.00±598.63	4705.33±1322.05	4663.83±662.61	3375.33±710.66
Segniliparaceae	675.00±309.68	503.17±307.20	483.17±267.96	2030.50±615.06	954.83±331.10	91.33±42.50	935.83±500.20
Shewanellaceae	1331.33±747.11	289.50±85.16	204.50±129.76	110.33±70.04	57.33±17.20	185.83±133.05	299.50±153.48
Thermoanaerobaculaceae	5742.83±1972.63	4474.17±1296.56	3335.83±795.82	4144.67±745.30	4740.83±1035.71	6196.83±1713.98	4354.33±1090.91
Vibrionaceae	1242.00±790.86	899.67±401.99	498.00±205.78	524.67±327.99	986.50±412.33	378.00±107.59	443.17±203.52
Woeseiaceae	2365.50±734.01	2097.17±538.11	2886.50±720.83	3637.00±709.35	3977.83±797.39	3422.00±1111.46	2672.17±631.94
